# SARS Coronavirus nsp1 Protein Induces Template-Dependent Endonucleolytic Cleavage of mRNAs: Viral mRNAs Are Resistant to nsp1-Induced RNA Cleavage

**DOI:** 10.1371/journal.ppat.1002433

**Published:** 2011-12-08

**Authors:** Cheng Huang, Kumari G. Lokugamage, Janet M. Rozovics, Krishna Narayanan, Bert L. Semler, Shinji Makino

**Affiliations:** 1 Department of Microbiology and Immunology, The University of Texas Medical Branch, Galveston, Texas, United States of America; 2 Department of Microbiology and Molecular Genetics, School of Medicine, University of California, Irvine, California, United States of America; University of North Carolina at Chapel Hill, United States of America

## Abstract

SARS coronavirus (SCoV) nonstructural protein (nsp) 1, a potent inhibitor of host gene expression, possesses a unique mode of action: it binds to 40S ribosomes to inactivate their translation functions and induces host mRNA degradation. Our previous study demonstrated that nsp1 induces RNA modification near the 5′-end of a reporter mRNA having a short 5′ untranslated region and RNA cleavage in the encephalomyocarditis virus internal ribosome entry site (IRES) region of a dicistronic RNA template, but not in those IRES elements from hepatitis C or cricket paralysis viruses. By using primarily cell-free, *in vitro* translation systems, the present study revealed that the nsp1 induced endonucleolytic RNA cleavage mainly near the 5′ untranslated region of capped mRNA templates. Experiments using dicistronic mRNAs carrying different IRESes showed that nsp1 induced endonucleolytic RNA cleavage within the ribosome loading region of type I and type II picornavirus IRES elements, but not that of classical swine fever virus IRES, which is characterized as a hepatitis C virus-like IRES. The nsp1-induced RNA cleavage of template mRNAs exhibited no apparent preference for a specific nucleotide sequence at the RNA cleavage sites. Remarkably, SCoV mRNAs, which have a 5′ cap structure and 3′ poly A tail like those of typical host mRNAs, were not susceptible to nsp1-mediated RNA cleavage and importantly, the presence of the 5′-end leader sequence protected the SCoV mRNAs from nsp1-induced endonucleolytic RNA cleavage. The escape of viral mRNAs from nsp1-induced RNA cleavage may be an important strategy by which the virus circumvents the action of nsp1 leading to the efficient accumulation of viral mRNAs and viral proteins during infection.

## Author Summary

Severe acute respiratory syndrome (SARS) coronavirus (SCoV) is the causative agent of SARS. The nsp1 protein of SCoV blocks host protein synthesis, including type I interferon, a general inhibitor of virus replication, in infected cells. This finding suggests that SCoV nsp1 protein plays a key role in the severe symptoms that accompany SARS infection. Nsp1 binds to the 40S ribosome subunit, which is an essential component for protein synthesis, and inactivates the translation activity of the ribosome. Furthermore, nsp1 binding to the 40S ribosome induces the modification of host mRNAs, leading to the accelerated decay of these RNAs in SCoV-infected cells. We found that the nature of nsp1-induced RNA modification was RNA cleavage and that nsp1 did not recognize specific nucleotides in host mRNAs to induce this cleavage. Interestingly, nsp1 did not induce RNA cleavage in SCoV mRNAs. These data indicate that nsp1 induces RNA cleavage of host mRNAs to suppress the expression of host genes, including those having antiviral functions; yet viral mRNAs are spared from such cleavage events, which, most likely, facilitate efficient SCoV protein synthesis and virus replication in infected cells.

## Introduction

Severe acute respiratory syndrome (SARS) coronavirus (SCoV) is the causative agent of SARS, which was first recognized in southern China in 2002 and spread to different areas of the world in a 2002-2003 epidemic [1]-[3]. It is believed that the bat-derived SCoV-like CoV [4], [5] underwent several mutations enabling the virus to cross the species barrier and replicate efficiently in humans [6]. Although it is uncertain whether SCoV-like CoV will re-emerge in the human community and initiate another SARS epidemic, the previous SARS outbreak made it apparent that CoVs, which usually cause only mild or moderate self-limiting symptoms in healthy humans, can cause a severe epidemic disease in our communities.

**Figure 1:**
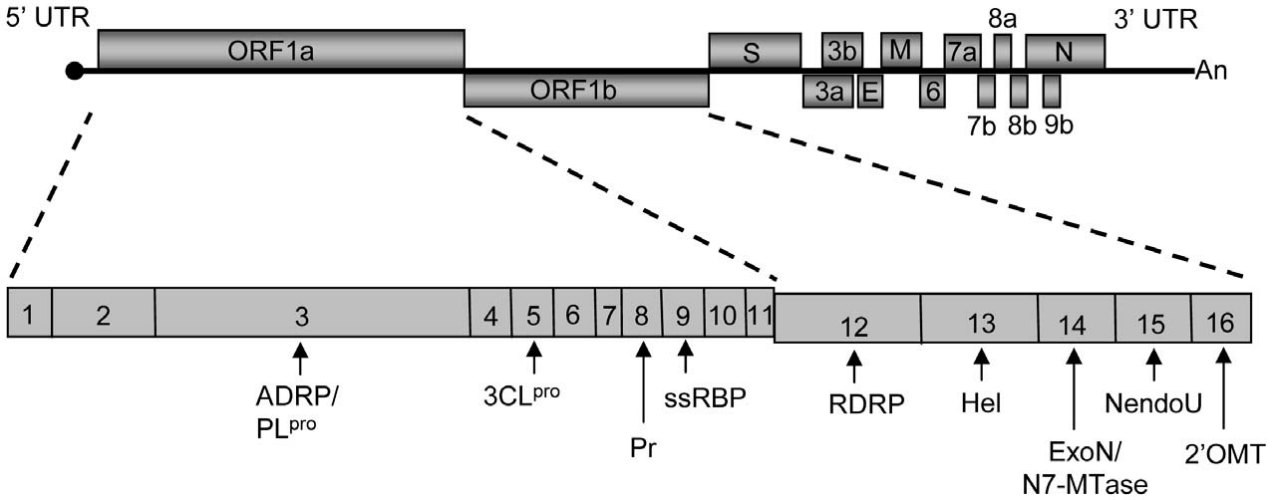
Schematic diagram of the SCoV genome. The viral genome consists of a single-stranded, positive-sense RNA of ∼29.7 kb in length. The 5′-proximal gene 1 (∼22-kb) has two ORFs, ORF1a and 1b, which encodes for large polyproteins, polyprotein 1a and polyprotein 1ab. The polyproteins are cleaved into 16 mature non-structural proteins (nsp1-nsp16) by viral proteases PL^pro^ and 3CL^pro^. Most of the non-structural proteins are involved in viral RNA synthesis and some of the identified functions of the non-structural proteins are: ADRP, ADP-ribose-1”-phosphate phosphatase [14]; PL^pro^, papain-like protease [72]; 3CL^pro^, 3C-like protease [73]; Pr, primase [11]; ssRBP, single-stranded RNA-binding protein [74]; RDRP, RNA-dependent RNA polymerase [9]; Hel, helicase [16]; ExoN, 3′5′ exoribonuclease; N7-MTase, guanine-N7-methyltransferase [69]; NendoU, poly(U)-specific endoribonuclease [8]; and 2′OMT, 2′ O-methyltransferase [68]. Major viral structural proteins, including S, M, N and E, and the accessory proteins, including 3a, 3b, 6, 7a, 7b, 8a, 8b, and 9b, are encoded downstream of the ORF1a/1b.

SCoV, which belongs to the betaCoV genus among the alpha, beta and gammaCoV genera in the family *Coronaviridae*, is an enveloped RNA virus carrying a long (∼30 kb), single-stranded, positive-sense genomic RNA. The 5′-proximal ∼22 kb-long gene 1 region of SCoV genomic RNA has two partially overlapping open reading frames (ORFs) 1a and 1b (Figure 1). Immediately after infection, the genomic RNA is translated to produce two large polyproteins; one is from ORF1a and the other from ORF1a and 1b via a ribosomal frame-shift mechanism [6], [7]. These two polyproteins are processed by two viral proteinases to generate 16 mature proteins, nsp1-nsp16 (Figure 1). Most of the gene 1 proteins are involved in viral RNA synthesis [8]-[16], while some have other biological functions [17]-[21]. To carry out viral gene expression, nine species of virus-specific mRNAs, including mRNA1, which is the intracellular form of genomic RNA, and eight species of subgenomic mRNAs, i.e., mRNA 2-mRNA 9, are produced in infected cells. These viral mRNAs make up a 3′-co-terminal, nested-set structure and accumulate in different quantities. Located at the 5′-end of all of these intracellular viral mRNAs and genomic RNAs is a ∼70 nt-long identical leader sequence.

SCoV nsp1 protein, which is the most N-terminal product of the gene 1 polyproteins, suppresses host gene expression in expressed cells and in infected cells [19], [22]. Nsp1 prevents type I interferon production in infected cells [23], and the expressed nsp1 suppresses the host antiviral signaling pathways [24]. Furthermore, the nsp1 of a closely related mouse hepatitis virus suppresses host gene expression, interferes with the type I interferon system, and is a virulence factor [25]. These data led us to suggest that SCoV nsp1 plays important roles in SARS pathogenesis. SCoV nsp1 suppresses host gene expression by using a novel, two-pronged strategy [19], [22]. Nsp1 binds to 40S ribosomes, leading to the inhibition of host protein synthesis. Ribosome-bound nsp1 further induces RNA modification of a capped mRNA, rendering it translationally incompetent [22]. Nsp1 protein promotes host mRNA degradation both in transiently transfected cells expressing nsp1 and in infected cells [19], [22], [23], [26]; cellular RNA decay functions most likely influence the efficient degradation of host mRNAs that undergo the nsp1-induced modification. The nsp1-induced RNA modification is template-dependent. Incubation of nsp1 in rabbit reticulocyte lysate (RRL) with a dicistronic RNA transcript harboring the encephalomyocarditis virus (EMCV) internal ribosome entry site (IRES) between two reporter genes results in RNA cleavage near the 3′-region of the EMCV IRES element, whereas nsp1 does not induce RNA cleavage in the IRES region of dicistronic RNA transcripts containing either the hepatitis C virus (HCV) IRES or the cricket paralysis virus (CrPV) IRES [22]. The molecular basis for the nsp1-mediated selective endonucleolytic RNA cleavage among these IRESes is unclear. Incubation of capped and polyadenylated reporter mRNA encoding the Renilla luciferase (rluc) gene with nsp1 in RRL and subsequent primer extension analysis of the reporter mRNA showed that the nsp1 induces several premature primer extension termination signals near the 5′-end of the mRNA [22]. Neither the nature of the nsp1-induced modification of capped mRNA nor the mechanism of the RNA modification site selection is known. Also unknown is the effect of nsp1 on SCoV mRNAs. Similar amounts of virus-specific mRNAs are detected in SCoV-infected cells and in cells infected with a SCoV mutant, SCoV-mt, which encodes the nsp1-mt protein carrying K164A and H165A mutations [23]. This mutated form of nsp1 neither binds to 40S ribosome subunits [22] nor promotes mRNA degradation [23], which suggests that SCoV mRNAs may escape from the nsp1-induced mRNA modification.

The present study was undertaken to clarify the nature of the nsp1-induced modification in capped mRNAs, explore the basis of the RNA modification site selection, characterize the template-dependent properties of the nsp1-induced RNA modification, and examine the effect(s) of nsp1 on the integrity of SCoV mRNAs primarily by using cell-free *in vitro* assays. Our data demonstrate that endonucleolytic RNA cleavage was the nature of the nsp1-induced modification of RNA templates, and RNAs carrying selective groups of IRESes were susceptible to the nsp1-induced RNA cleavage. The contribution of RNA secondary structures of template mRNAs for the selection of the RNA cleavage sites is also suggested. Finally, we discovered that SCoV mRNAs were resistant to the nsp1-induced RNA modification, a finding suggesting that SCoV has developed a strategy to selectively protect its own mRNAs from nsp1-induced RNA modifications to ensure efficient viral gene expression during infection.

## Results

### Susceptibilities of dicistronic mRNAs carrying different IRESes to nsp1-induced RNA cleavage

SCoV nsp1 induces endonucleolytic RNA cleavage near the 3′-region of the EMCV IRES of dicistronic RNA transcripts, Ren-EMCV-FF, in which expression of the upstream rluc ORF is mediated by cap-dependent translation and the translation of downstream firefly luciferase (fluc) ORF is driven by the EMCV IRES in both RRL [22] and cultured cells [27]. In contrast, SCoV nsp1 does not induce RNA cleavage in similar dicistronic RNA transcripts carrying the HCV IRES or the CrPV IRES between rluc and fluc genes in RRL [22]. EMCV, HCV and CrPV belong to the picornavirus family, flavivirus family and dicistrovirus family, respectively. Although currently divided into four distinct categories [28], picornavirus IRES elements were originally grouped into type I and type II IRESes based on their primary sequence and secondary structure similarities [29]. IRES elements within the same IRES group display a high homology in RNA secondary structures, but only modest similarity in their primary sequences, while IRESes from different groups have distinct RNA secondary structures. Picornavirus type I IRESes include IRESes derived from poliovirus, coxsackie B virus (CVB) and human rhinovirus (HRV), while picornavirus type II IRESes include those derived from EMCV and Theiler's murine encephalomyelitis (TMEV). Because HCV, CrPV and picornavirus type I and type II IRESes are distinct in terms of their primary sequences, secondary structures and requirements for translation initiation factors (for review refer to [30]-[32]), testing the susceptibilities of these IRESes to nsp1-induced endonucleolytic RNA cleavage would provide a clue towards understanding the role of RNA secondary structures and host translation initiation factors in the nsp1-induced RNA cleavage.

**Figure 2:**
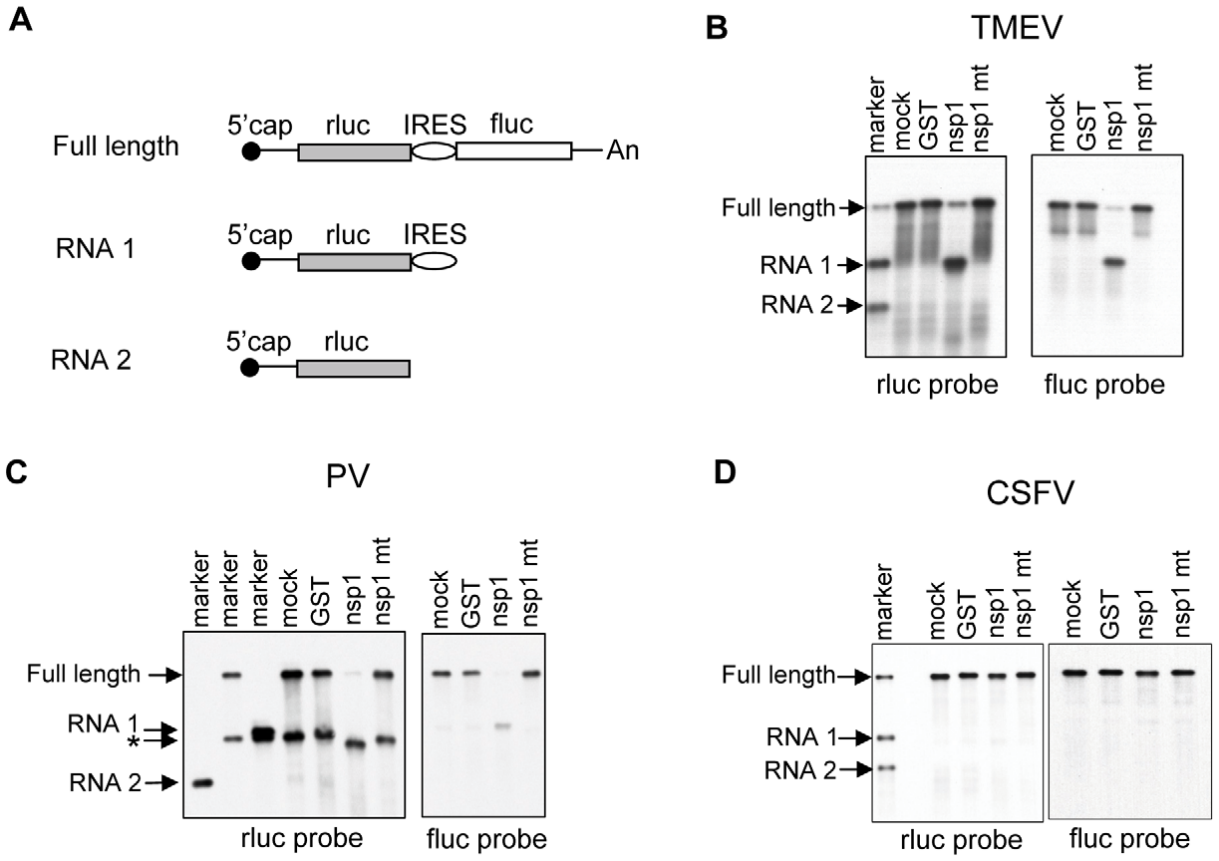
Susceptibilities of IRES-containing dicistronic RNAs to nsp1-mediated RNA cleavage. (A) Schematic diagram of the structures of full length dicistronic RNA transcripts (Full length), RNA 1 containing the 5′ rluc gene and intercistronic IRES sequence and RNA 2 containing only the 5′ rluc gene. (B) Ren-TMEV-FF was incubated with GST, nsp1 or nsp1-mt or without any protein (mock) in RRL at 30^o^C for 10 min. RNA samples were extracted and analyzed by Northern blot analysis using the 5′ rluc probe (left panel) and 3′ fluc probe (right panel). Marker is a mixture of *in vitro*-transcribed, full-length RNA transcripts, RNA 1 and RNA 2. (C) Ren-PV-FF was examined as in (B), except RNA was incubated in RRL+HeLa. RNA 2, full length dicistronic RNA transcripts and RNA 1 were separately applied to the gel and shown in three marker lanes. An asterisk represents a truncated RNA product, which was probably generated by premature transcription termination around the IRES region of full-length RNA and RNA 1. (D) Ren-CSFV-FF was examined as described in (B).

To determine the molecular basis for the nsp1-induced, template-dependent endonucleolytic RNA cleavage, we tested whether nsp1 induced RNA cleavage in the IRES region of a series of dicistronic RNA transcripts, each containing an IRES derived from different picornaviruses, including TMEV (Ren-TMEV-FF), poliovirus (Ren-PV-FF), CVB (Ren-CVB-FF), and HRV 2 (Ren-HRV2-FF) or a flavivirus, classical swine fever virus (CSFV) (Ren-CSFV-FF); the latter IRES has an HCV-like IRES structure. In all transcripts, expression of the upstream rluc ORF was mediated by cap-dependent translation and the translation of downstream fluc ORF driven by the IRES. The Ren-TMEV-FF or Ren-CSFV-FF transcripts were incubated in RRL with a recombinant nsp1 protein, which was initially expressed as glutathione S-transferase (GST)-nsp1 fusion protein in *E. coli*. The GST tag was subsequently eliminated [19]. For analysis of Ren-PV-FF, Ren-CVB-FF and Ren-HRV2-FF transcripts, RRL containing 20% (vol/vol) HeLa S10 extract [33] (RRL+HeLa) was used; translation activities of these picornavirus-derived IRESes require host factors that are missing or exist in low abundance in RRL [34]-[36]. Thus, RRL+HeLa is used for translation mediated by these IRESes [37]. As controls, the RNA was left untreated or incubated with a non-specific control protein,GST or a mutated form of nsp1, nsp1-mt with K164A and H165A mutations [23]. Nsp1-mt does not bind to 40S ribosomes and lacks the translational suppression and template RNA modification activities [19], [23]. After incubation, RNAs were extracted and subjected to Northern blot analysis by using an rluc probe hybridizing to the rluc ORF and a fluc probe hybridizing to the fluc ORF (Figure 2 and Figure S1). To estimate the RNA cleavage sites, we included three RNA size markers for each template RNA; they were an untreated template (full length), RNA 1 containing the region from the 5′-end to the 3′-end of the inter-cistronic region of the template, and RNA 2 containing the region from the 5′-end to the end of the rluc ORF of the template (Figure 2A). As expected, incubation of all RNA transcripts with GST or nsp1-mt did not induce RNA cleavage (marked as GST and nsp1 mt in Figure 2 and Figure S1). Incubation of Ren-TMEV-FF with nsp1 resulted in reduction of full-length transcript abundance and generation of two major RNA fragments (Figure 2B). The size of the 5′-fragment, which was detected by the rluc probe, indicated that nsp1 induced an endonucleolytic cleavage near the 3′-end of TMEV IRES. All RNA transcripts carrying picornavirus type I IRES, including Ren-PV-FF, Ren-CVB-FF and Ren-HRV2-FF, underwent the nsp1-induced RNA cleavage (Figure 2C, Figures S1B and S1C). Ren-PV-FF preparations contained an unexpected RNA band, which was detected by the rluc probe and was slightly smaller than the RNA 1 marker (denoted by the asterisk in Figure 2C), leading us to suggest that this RNA was generated by premature transcriptional termination near the 3′ end of the poliovirus IRES. The 5′ fragment of dicistronic transcripts carrying picornavirus type II IRES and the RNA 1 markers showed a similar migration in the gel (Figure 2B) [22], whereas the corresponding RNA fragment of dicistronic transcripts carrying a picornavirus type I IRES migrated slightly faster than did the RNA 1 markers in the gels; the size difference between the 5′ fragment of Ren-HRV2-FF and the RNA 1 maker was less prominent than those between the 5′ fragment of Ren-PV-FF or Ren-CVB-FF and their RNA 1 makers (Figure 2 and Figures S1B and S1C). In contrast to dicistronic transcripts carrying picornavirus type I IRES or type II IRES, nsp1 did not induce RNA cleavage in Ren-CSFV-FF (Figure 2D).

We previously reported that co-transfection of a plasmid encoding nsp1 and one that encoded dicistronic RNA transcripts carrying the EMCV IRES resulted in RNA cleavage of the expressed dicistronic RNA transcripts, demonstrating that expressed nsp1 exerts RNA cleavage in cultured cells [27]. The finding of RNA cleavage following co-expression in cultured cells of nsp1, but not nsp1-mt or chloramphenicol acetyltransferase (CAT), with dicistronic RNA transcripts carrying the TMEV IRES or poliovirus IRES, but not those carrying the HCV IRES or CSFV IRES, (Figure S2) demonstrated that expressed nsp1 exerted template-dependent endonucleolytic RNA cleavage. In addition, nsp1 expression reduced the abundances of the full-length RNAs of all of the expressed RNA transcripts. Because the nsp1 induces modification at the 5′ region of the capped RNA transcripts in RRL [22], we suspect that expressed RNAs most likely underwent nsp1-induced modification near the 5′ end and were degraded by host mRNA decay functions, resulting in the reduction of the abundance of the expressed RNA transcripts in nsp1-expressing cells. In summary, nsp1 induced endonucleolytic RNA cleavage in RNA transcripts carrying the IRESes of picornaviruses, but not in those carrying CSFV IRES, both *in vitro* and *in vivo*.

### Identification of the endonucleolytic RNA cleavage sites in Ren-EMCV-FF and Ren-PV-FF

**Figure 3:**
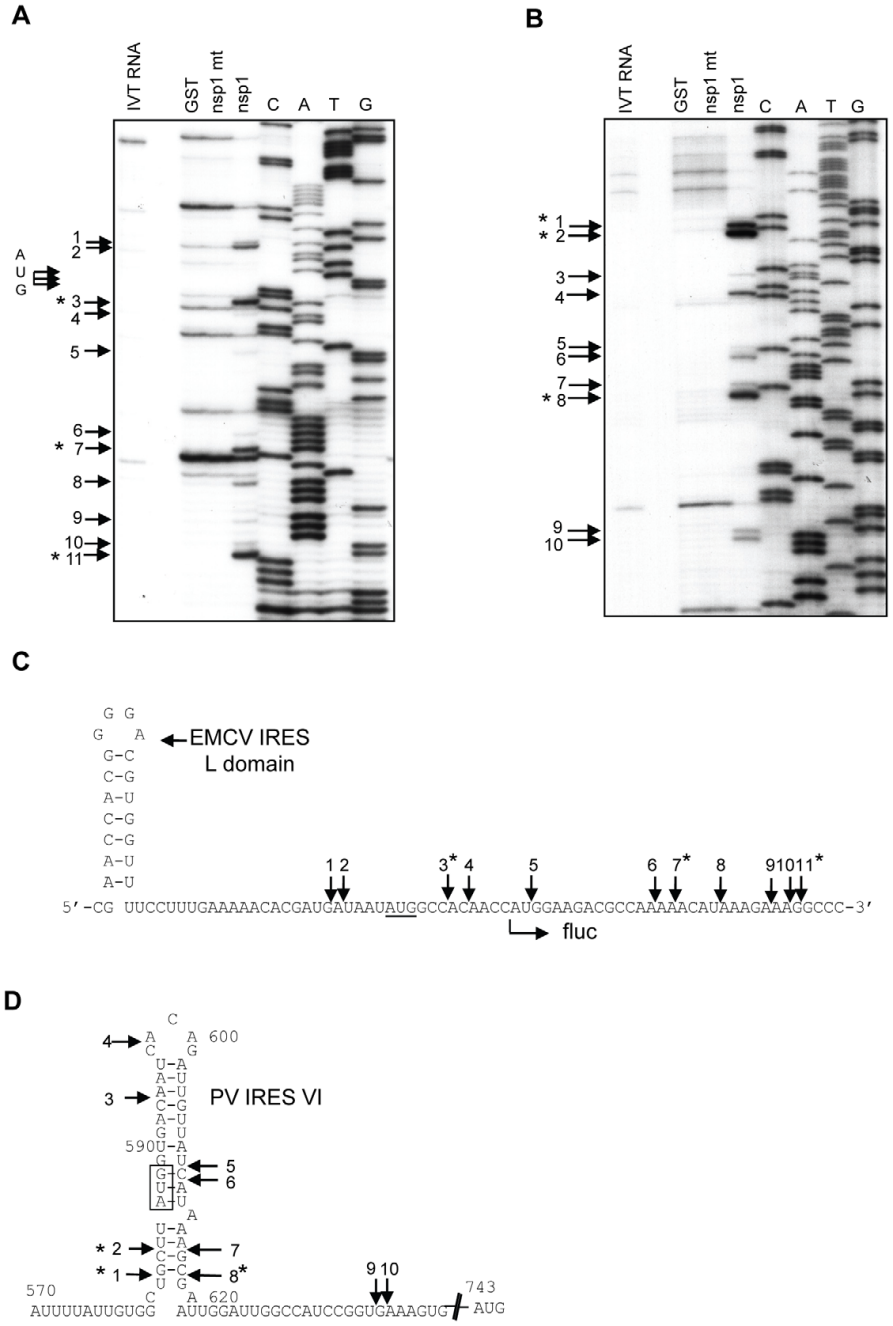
Primer extension analysis of Ren-EMCV-FF and Ren-PV-FF after incubation with GST, nsp1 or nsp1-mt. (A, B) *In vitro*-synthesized Ren-EMCV-FF (A) and Ren-PV-FF (B) were incubated with GST, nsp1 or nsp1-mt in RRL (A) and RRL+HeLa (B), respectively. The RNAs were extracted and subjected to primer extension analysis by using the 5′-end ^32^P labeled primer that binds at ∼100 nt downstream of the fluc translation initiation codon for Ren-EMCV-FF (A) or a labeled primer that binds 6 nt downstream of the fluc initiation codon region for Ren-PV-FF (B). Primer extension products and DNA sequence ladders, which were generated from the plasmid used for RNA synthesis and the primer used for primer extension analysis, were resolved on 8% polyacrylamide/7M Urea DNA sequencing gels. Premature primer extension termination signals are indicated by enumerated arrows and major RNA modification sites are marked with asterisks. IVT RNA, primer extension analysis of *in vitro*-transcribed RNAs that were not incubated in RRL; AUG, translation initiation AUG (AUG-834) of EMCV IRES. (C, D) RNA modification sites in Ren-EMCV-FF (C) and Ren-PV-FF (D). The structural domain L of the EMCV IRES (C) [39] in Ren-EMCV-FF and the poliovirus IRES stem-loop IV (D) [75] in Ren-PV-FF are also shown. Arrows indicate RNA modification sites and asterisks denote the main ones. The underlined AUG triplet is equivalent to the translation initiation codon AUG-834 in the EMCV genome (C) and the boxed AUG triplet is equivalent to the AUG-586 of the poliovirus genome. AUG-586 of the poliovirus is mapped ∼150 nt upstream of the authentic viral translation initiation codon.

We next determined the nsp1-induced RNA cleavage sites in Ren-EMCV-FF and Ren-PV-FF RNA. We took advantage of the fact that the RNA structure as well as the structural and functional relationships of the EMCV IRES and the poliovirus IRES are well characterized [38], [39]. Ren-EMCV-FF that had been incubated with GST, nsp1 or nsp1-mt in RRL was subjected to primer extension analysis using the 5′-end labeled primer that binds at a site ∼100 nt downstream of the fluc gene initiation codon. Three strong (sites 3, 7 and 11) and several minor primer extension termination signals were detected in the sample that was incubated with nsp1, but not with GST or nsp1-mt (Figure 3A). Site 3 was located 3-nt downstream of the translation initiation AUG (AUG-834) of the EMCV IRES (underlined AUG in Figure 3C), while sites 7 and 11 were located within the 5′ region of the fluc ORF. Two minor primer extension termination sites 1 and 2 existed upstream of AUG-834 and other minor sites were detected between AUG-834 and site 11. Previous studies reported the possibility that the 43S pre-initiation complex, which is made up with 40S ribosome, eIF1, eIF1A, ternary complex (eIF2, Met-tRNA, and GTP), and eIF3, loads onto the EMCV IRES at or near the AUG-834 [40], [41]. Hence, our data may indicate that nsp1 induced several endonucleolytic RNA cleavages at or in the proximity of the ribosome loading site of the EMCV IRES of Ren-EMCV-FF.

Northern blot analysis indicated that the nsp1 induced the RNA cleavage roughly 100-200 nt upstream of the initiation codon of the downstream fluc ORF of Ren-PV-FF (Figure 2C); hence, for primer extension analysis of Ren-PV-FF we used a primer that binds at a site 6 nt downstream of the translation initiation codon of the fluc ORF. Primer extension analysis of Ren-PV-FF that had been incubated with nsp1, but not with GST or nsp1-mt, in RRL+HeLa revealed three major extension termination signals, namely sites 1, 2 and 8, near an AUG (AUG-586), which corresponds to the AUG at 586 nt of the poliovirus genome, located within the poliovirus IRES domain VI (Figures 3B and 3D). All three major sites 1, 2 and 8, and a minor site 7 were located in close proximity within the computer-predicted secondary structure of poliovirus IRES domain VI (Figure 3D). Other minor primer extension termination sites were located downstream of AUG-586, which is considered to be a part of ribosome binding site within poliovirus IRES. It should be noted that AUG-586 is not used for viral translation initiation [42]; viral translation initiates from another AUG triplet (AUG-743) located ∼150 nt downstream of this silent AUG-586 by ribosome shunting or scanning mechanisms [42]-[44]. In the Ren-PV-FF transcripts, AUG-743 served as the translation initiation codon for the fluc gene. Judging from the migration of the 5′ RNA cleavage product of the Ren-PV-FF relative to marker RNA 1, which is an RNA fragment corresponding to the 5′-end of Ren-PV-FF to 30-nt downstream of AUG-743 (Figure 2C), the size of the 5′ RNA fragment of Ren-PV-FF and the major RNA cleavage sites 1, 2 and 8 in Ren-PV-FF were in good agreement. We did not detect major primer extension termination products near AUG-743 following the use of another primer that binds ∼100 nt downstream of the AUG-743 (data not shown). These data strongly suggested that nsp1 induced RNA cleavage at or near the 40S ribosome loading site within poliovirus IRES of Ren-PV-FF.

### Characterization of the SCoV nsp1-induced RNA modification of capped non-viral mRNAs

**Figure 4:**
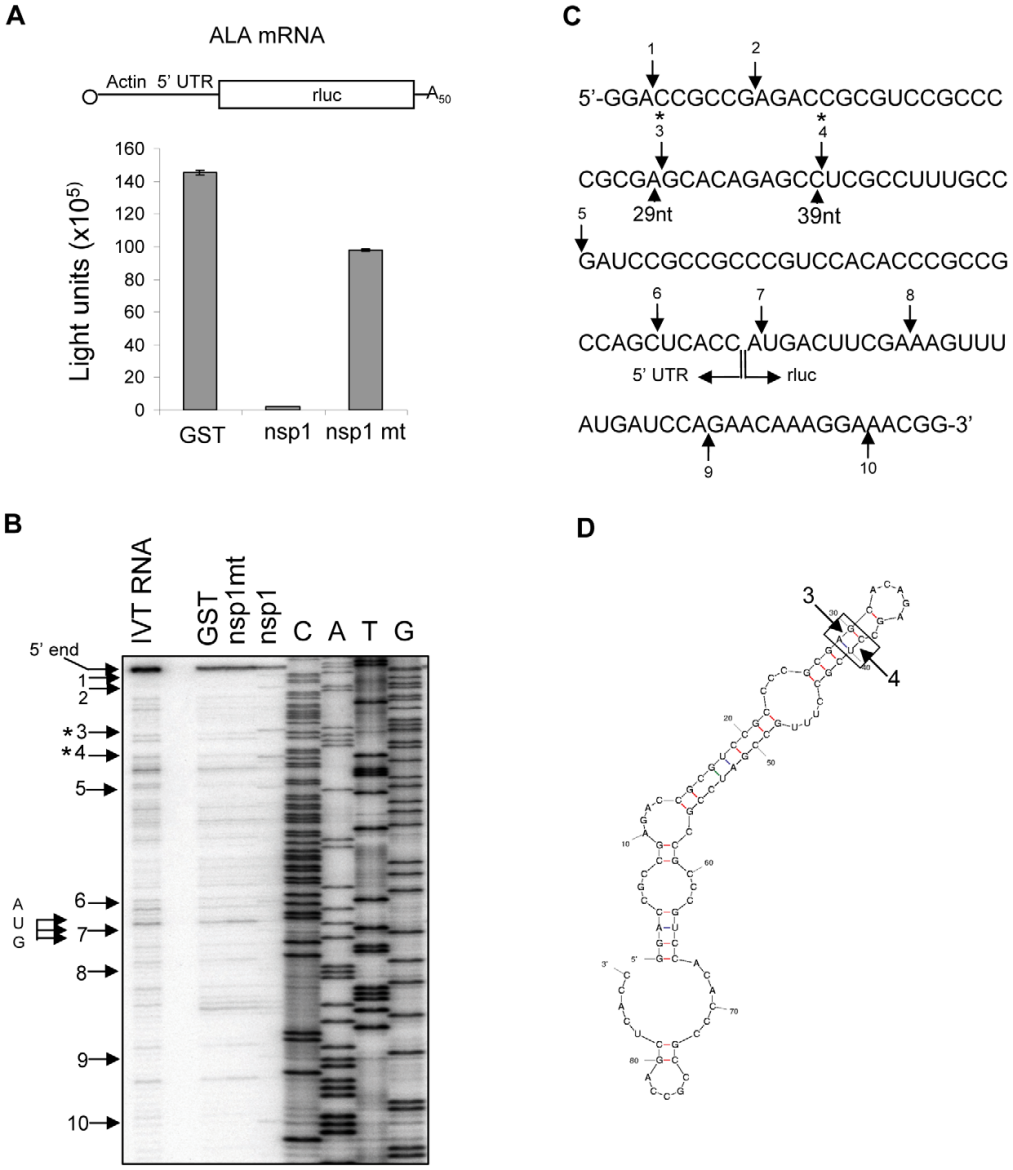
Characterization of nsp1-induced RNA modification in ALA mRNA. (A) Capped and polyadenylated ALA mRNA was incubated with GST, nsp1 or nsp1-mt in RRL, and rluc reporter activities were determined by an rluc assay (Promega). At the top is a schematic diagram of ALA mRNA, in which 5′ UTR and the rluc gene are not shown to scale. (B) RNAs were extracted after incubation with GST, nsp1 or nsp1-mt and subjected to primer extension analysis by using a 5′-end labeled primer that binds to ∼100 nt downstream of the rluc gene translation initiation codon. Primer extension products were resolved on 8% polyacrylamide/7M Urea DNA sequencing gels, and premature primer extension termination signals are shown by enumerated arrows. Main RNA modification products (sites 3 and 4) are marked with asterisks. 5′-end, full-length primer extension product; IVT RNA, primer extension analysis of *in vitro*-transcribed ALA mRNA that was not incubated in RRL; AUG, translation initiation codon of rluc gene. (C) RNA modification sites of ALA mRNA. Arrows indicate RNA modification sites with main RNA modification sites marked with asterisks. (D) Predicted secondary structure of human β-actin 5′ UTR. Arrows indicate main RNA modification sites.

**Figure 5:**
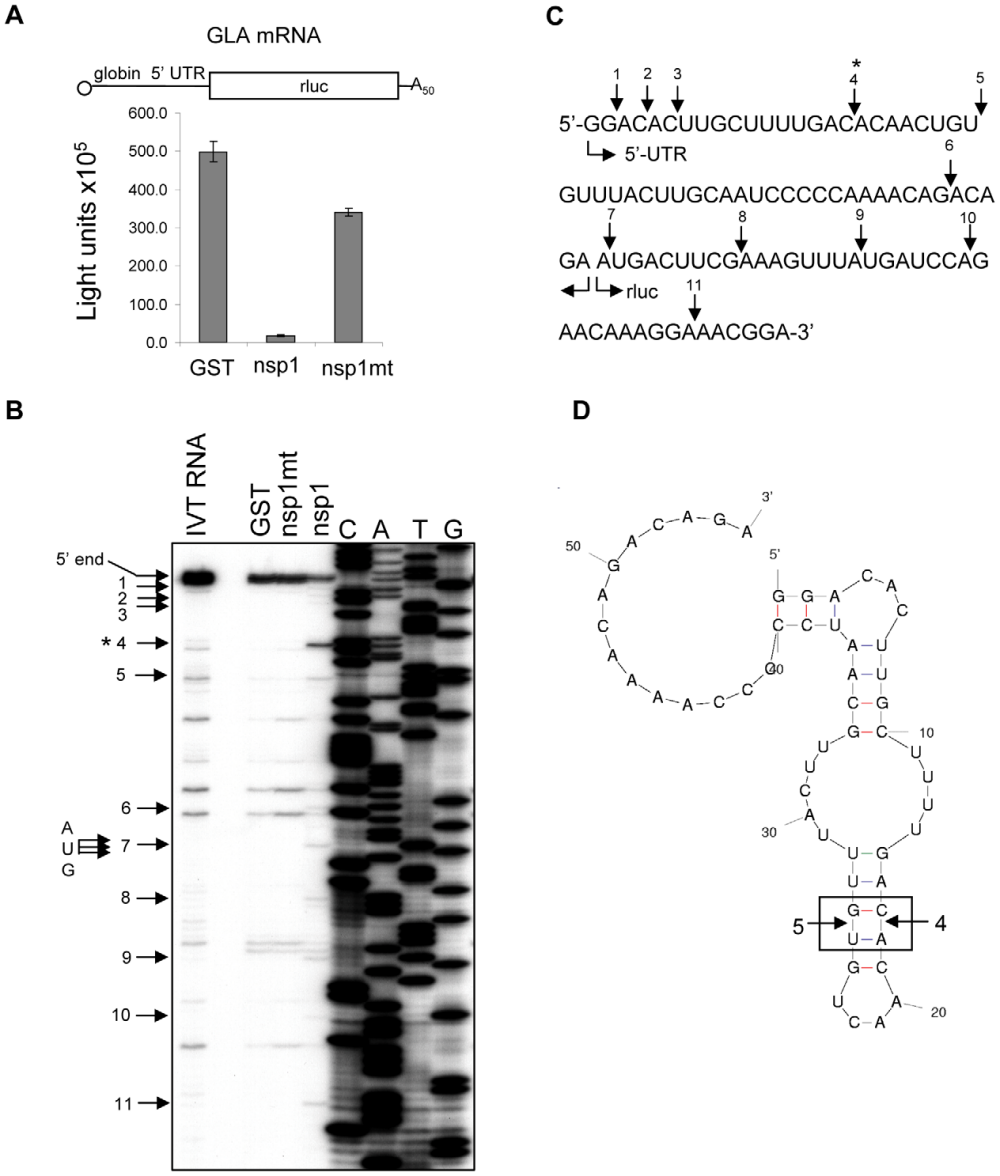
Characterization of nsp1-induced RNA modification in GLA mRNA. (A-D) Experiments similar to those described in Figure 4 were performed to obtain the results depicted in Figure 5, except that GLA mRNA, instead of ALA mRNA, was used.

Our previous studies used rluc RNA, which is a capped and polyadenylated mRNA encoding the rluc gene, as a model mRNA template for characterizing the nsp1-induced capped mRNA modification [22]. Incubation of rluc RNA with nsp1 in RRL generated several premature primer extension termination products indicative of cleavage near the 5′-end of rluc RNA, and the modified rluc RNA became translationally inactive [22]. The nature of the nsp1-induced modification, which causes primer extension terminations, in the rluc RNA is unknown. The rluc RNA has a short, 5′ untranslated region (UTR) of only 11 nt, which is atypical for most host mRNAs which have 5′ UTRs ranging from 20-100 nt in length [45]. Rabbit globin mRNA having a 53 nt-long 5′ UTR and β-actin mRNA carrying an 84 nt-long 5′ UTR are two of the host mRNAs widely utilized in molecular biology studies [46]-[50]. Thus, we used two *in vitro*-synthesized capped and polyadenylated mRNAs encoding the rluc gene which carried the 5′ UTR of human β-actin mRNA or that of rabbit β-globin mRNA to characterize nsp1-mediated RNA modification of capped, monocistronic mRNAs. Incubation of ALA mRNA (containing the β-actin 5′ UTR) or GLA mRNA (containing the rabbit β-globin 5′ UTR) with nsp1, but not GST or nsp1-mt, in RRL resulted in the efficient suppression of rluc protein expression (Figures 4A and 5A). Primer extension analysis of ALA mRNA, which was extracted from RRL after incubation with nsp1, but not with GST or nsp1-mt, showed two main premature primer extension termination products at nucleotides 29 (site 3) and 39 (site 4) (Figure 4B, indicated by asterisks) and several minor premature primer extension termination signals (Figure 4B, arrows). Incubation of GLA mRNA with nsp1, but not GST or nsp1-mt, resulted in a major premature primer extension termination product at nucleotide 17 (site 4) and several additional minor premature primer-extension termination sites (Figure 5B, arrows). The computer-assisted modeling of secondary structure [51] of the 5′ UTR of ALA mRNA showed a proximal location of sites 3 and 4 in a stem region of a stem-loop structure (Figure 4D). Likewise, a major RNA modification site 4 and a minor RNA modification site 5 of GLA mRNA were detected in close proximity to one another within a stem region of a predicted stem-loop structure (Figure 5D).

**Figure 6:**
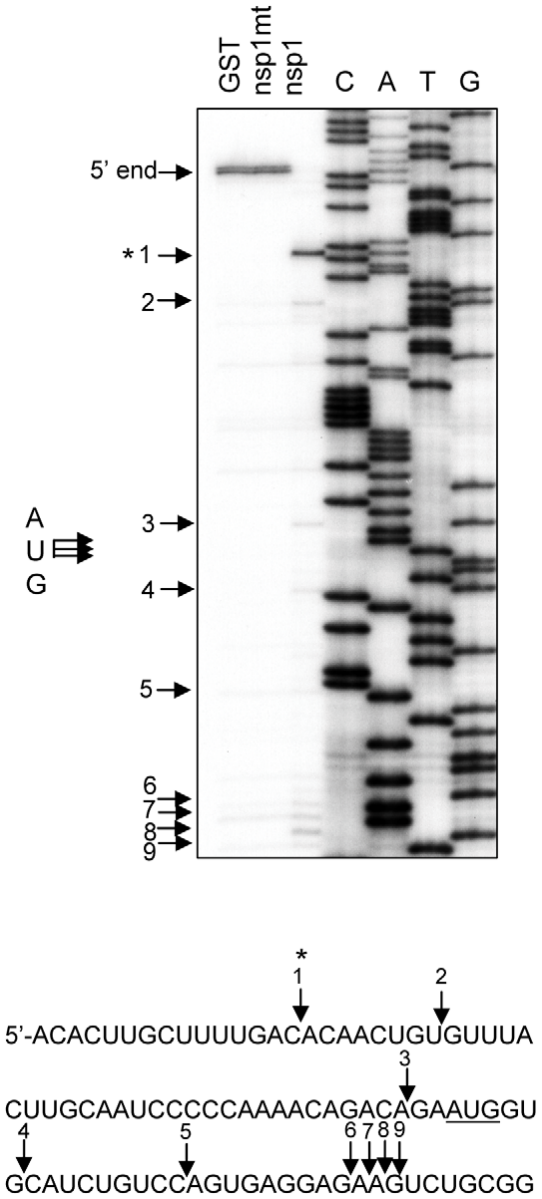
Characterization of nsp1-induced RNA modification in rabbit β-globin mRNA. Poly A+ RNA was extracted from micrococcal nuclease-untreated RRL (Promega) and incubated with GST, nsp1 or nsp1-mt in micrococcal nuclease-treated RRL. RNA was extracted and subjected to primer extension analysis with a rabbit β-globin mRNA-specific primer. Primer extension products were resolved on 8% polyacrylamide/7M Urea DNA sequencing gels (Top panel). Premature primer extension termination signals are shown by enumerated arrows. The main RNA modification product (site 1) is marked with an asterisk. The 5′-end represents a full-length primer extension product, and the AUG at the top of the figure and underlined AUG represent the translation initiation codon of β-globin mRNA.

To confirm that nsp1 also induces RNA modification in naturally occurring host mRNAs, rabbit β-globin mRNA obtained from RRL was incubated with GST, nsp1 or nsp1-mt in RRL, and the extracted RNA was subjected to primer extension analysis. A major premature extension termination site and ∼8 minor products were detected in the sample incubated with nsp1, but not nsp1-mt or GST (Figure 6). All three RNA modification sites within the 5′ UTRs of β-globin mRNA were also detected at the corresponding sites of the nsp1-treated GLA mRNA, whereas the most 5′-end minor modification sites 1-3 of GLA mRNA (Figures 5B and 5C) were barely detected in β-globin mRNA. The amount of the full-length primer extension product of the nsp1-treated β-globin mRNA was very low (Figure 6), which indicated that there was efficient nsp1-induced RNA modification in the naturally occurring β-globin mRNA.

**Figure 7:**
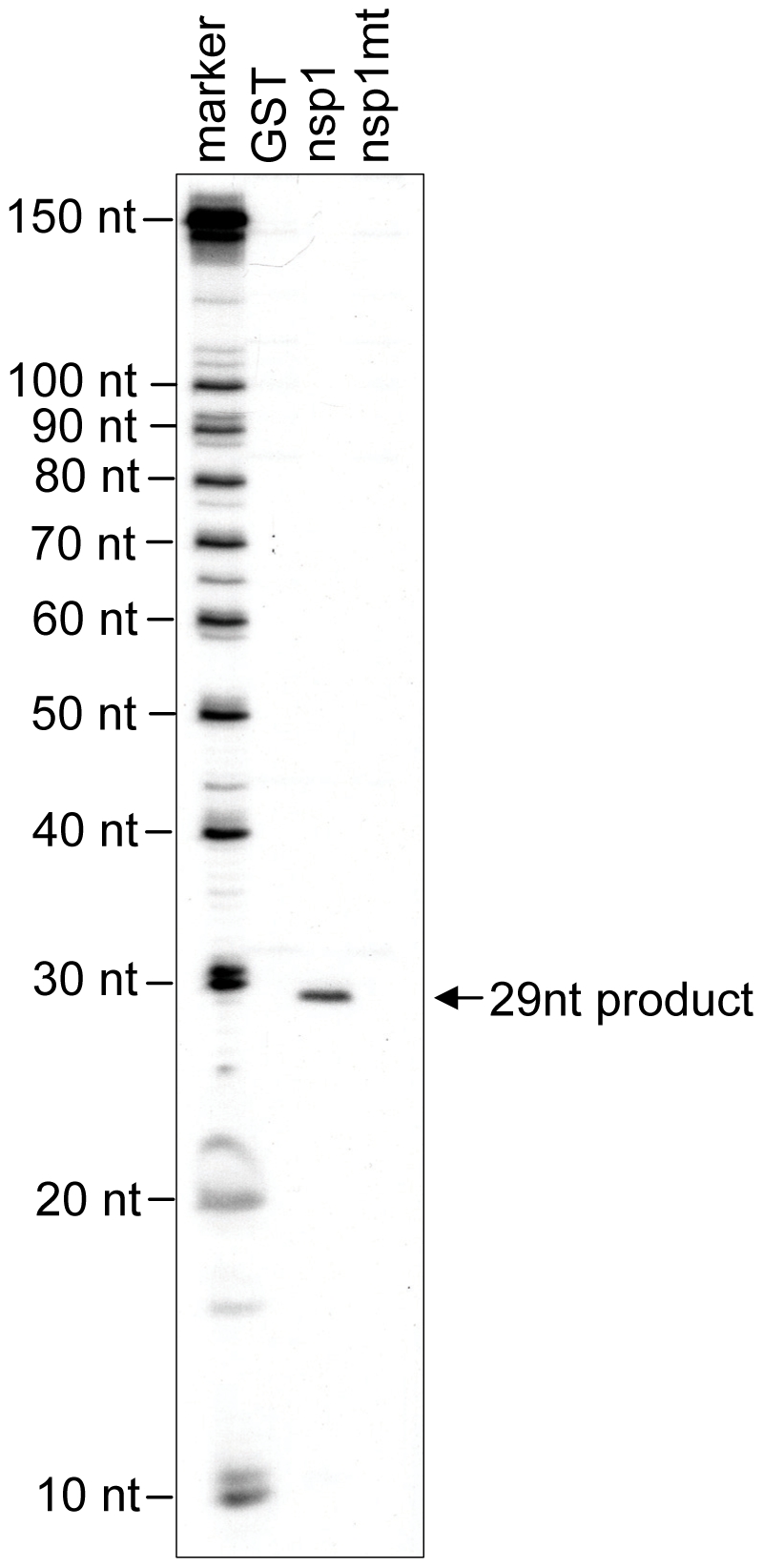
Characterization of nsp1-induced RNA fragment of ALA mRNA. Cap-labeled ALA mRNA was incubated with GST, nsp1 or nsp1-mt in RRL. RNAs were extracted from RRL by using proteinase K digestion and subsequent phenol/chloroform extraction. A cap-radiolabeled RNA fragment was detected in a 10% polyacrylamide/7M Urea DNA sequencing gel electrophoresis. An RNA size marker (ranging from 10 nt to 100 nt in 10 nt increment) was prepared by using the Decade Marker system (Applied Biosystems) and applied to the same gel.

To further clarify the nature of the nsp1-induced RNA modification of the capped mRNAs, ^32^P cap-labeled ALA mRNA, extracted after incubation with GST, nsp1 or nsp1-mt in RRL, was subjected to electrophoresis in a 10% polyacrylamide/8M urea sequencing gel. A major ∼29 nt RNA product was detected in the sample incubated with nsp1, but not with GST or nsp1-mt (Figure 7), which showed that nsp1 induced an endonucleolytic RNA cleavage in ALA mRNA. Notably, this endonucleolytic RNA cleavage product appeared to correspond to a major primer extension termination site 3 (Figure 4).

**Figure 8:**
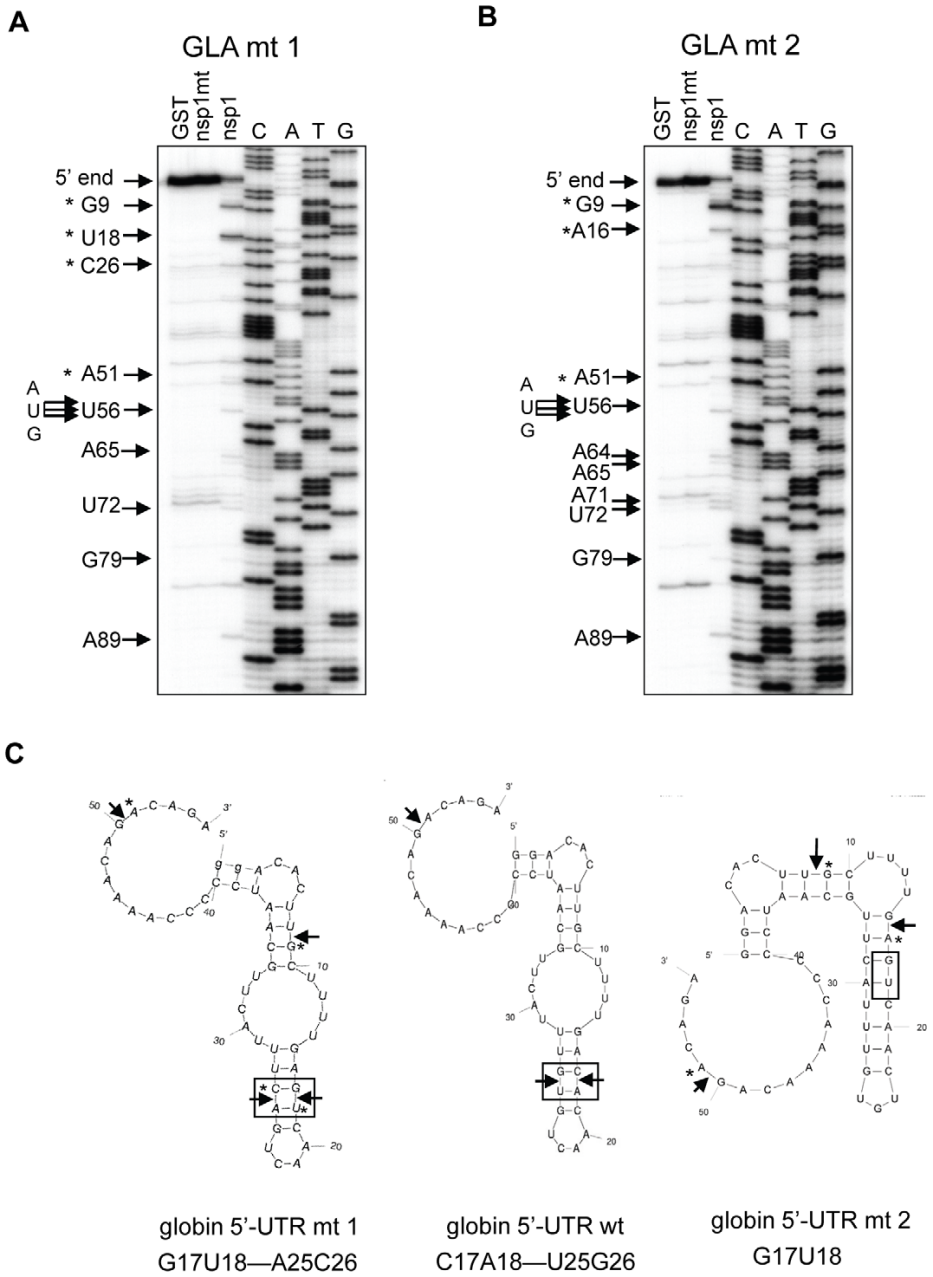
Identification of RNA modification sites of GLA mutant mRNAs. (A) GLA mutant 1 (GLA mt 1) carrying G17U18 and A25C26 mutations was incubated with GST, nsp1 or nsp1-mt in RRL. RNA was extracted from RRL and subjected to primer extension analysis. Premature primer extension termination signals are shown by enumerated arrows and asterisks represent RNA modification products in the 5′ UTR. 5′-end, full-length primer extension product; AUG, translation initiation codon of GLA mutant 1. (B) Similar experiments were performed by using GLA mutant 2 (GLA mt 2) with G17U18 mutations. (C) The predicted RNA secondary structures and the nsp1-induced RNA modification sites at the 5′ UTRs of GLA mutant 1 (left), GLA mRNA (middle) and GLA mutant 2 (right). Arrowheads show the RNA modification sites. Mutated sites in the mutants and the corresponding sites of GAL mRNA are boxed.

Experiments using Ren-EMCV-FF, Ren-PV-FF, GLA mRNA, β-globin mRNA, or ALA mRNA collectively showed that the nsp1 induced endonucleolytic RNA cleavages adjacent to any of the four nucleotides and between different di-nucleotide pairs, which may imply there is little or no preference for specific nucleotides at the RNA cleavage site. Computer-assisted RNA secondary structure analysis implicated a main RNA cleavage and another RNA cleavage occurring at highly proximal positions within stem-loop structures in the 5′ noncoding region of ALA, β-globin and GLA mRNAs and the 3′-region of the poliovirus IRES of Ren-PV-IRES transcripts. To clarify the importance of the di-nucleotide sequence around the cleavage site for the nsp1-induced RNA cleavage, we examined the nsp1-induced endonucleolytic RNA cleavage sites in a mutated GLA mRNA mt 1, which had the same predicted RNA secondary structure as that of GLA mRNA at the 5′ noncoding region and carried two nucleotide substitutions from C17A18 to G17U18 at the major cleavage site and another two nucleotide substitutions from U25G26 to A25C26; the latter two nucleotide substitutions were necessary to retain the predicted RNA secondary structure (refer to Figure 8C). Primer extension analysis showed that nsp1 induced a major endonucleolytic RNA cleavage at nucleotides 18 and 26 of GLA mt 1(Figure 8A), supporting the notion that there is little or no requirement for a specific nucleotide sequence at the RNA cleavage site for nsp1-induced RNA cleavage. Unlike GLA mRNA, GLA mRNA mt 1 had an additional cleavage site at nucleotide 9 (Figure 8A). Incubation of nsp1 with GLA mRNA mt 2 (carrying only a C17A18 to G17U18 change at the major cleavage site and having an altered predicted RNA secondary structure) in RRL resulted in major and minor cleavages at nucleotides 9 and 16, respectively (Figure 8B). These data led us to conclude that the nsp1-induced endonucleolytic RNA cleavage of the template mRNAs displayed no apparent nucleotide preference at the RNA cleavage site, while altering the RNA secondary structure affected the pattern of cleavage.

### Testing the susceptibility of SCoV mRNAs to nsp1-induced endonucleolytic RNA cleavage

**Figure 9:**
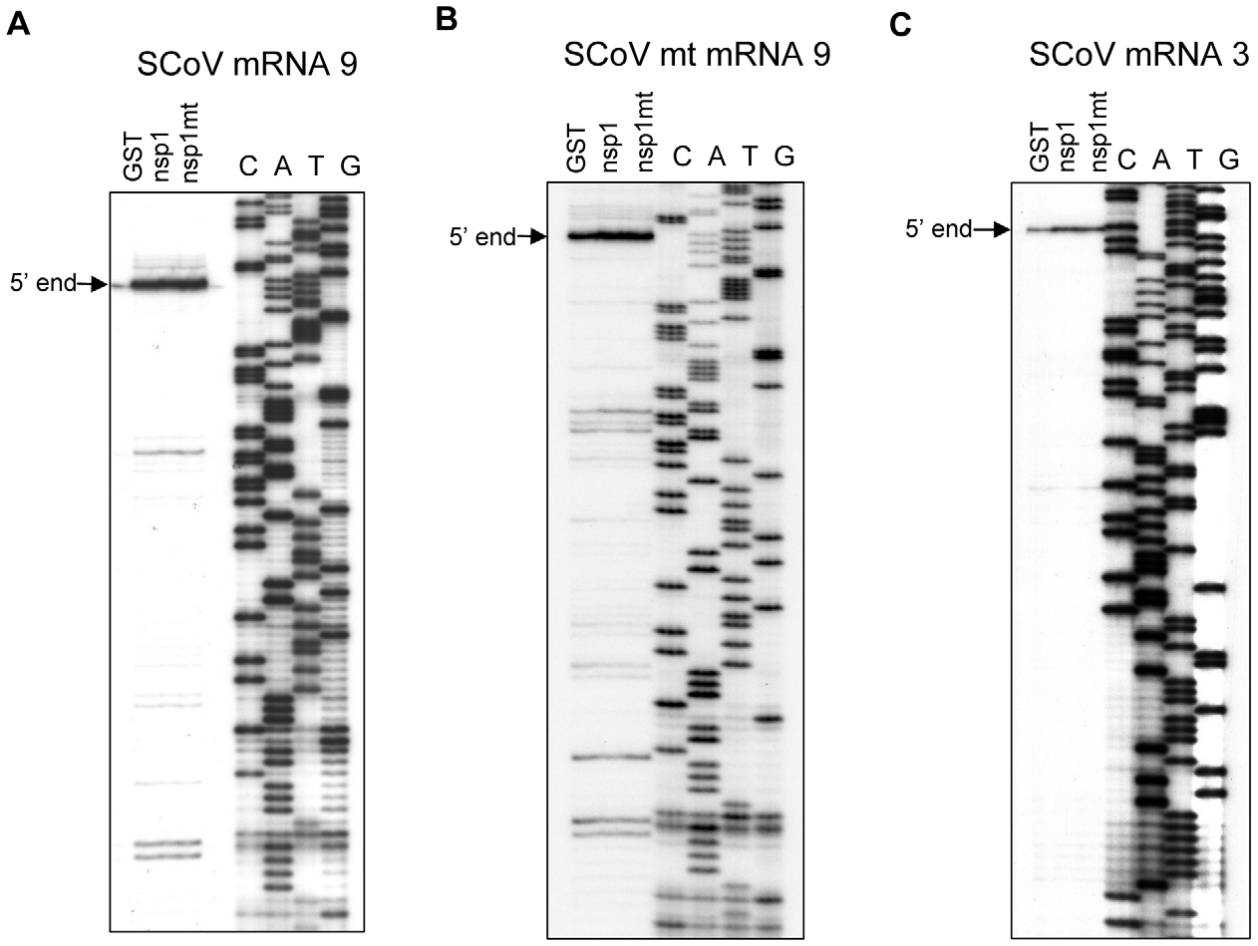
Susceptibilities of naturally occurring SCoV mRNAs to nsp1-induced RNA modification. (A and C) Poly A+ RNA was extracted from SCoV-infected cells and incubated with GST, nsp1 or nsp1-mt in RRL. Extracted RNAs were subjected to primer extension analysis by using primers suitable for detecting RNA modifications in SCoV mRNA 9 (A) or in SCoV mRNA 3 (C). Primer extension products were resolved in 8% polyacrylamide/7M Urea DNA sequencing gels: 5′-end, full-length primer extension product of SCoV mRNA 9 (A) or SCoV mRNA 3 (C). (B) Similar experiments described in (A) were performed, except that RNAs from SCoV-mt-infected cells were used.

Efficient viral gene expression occurs in SCoV-infected cells in spite of the nsp1-mediated host gene expression suppression [52]. Furthermore, cells infected with SCoV and those infected with SCoV-mt, a SCoV mutant encoding nsp1-mt, accumulated similar amounts of SCoV mRNAs [23]. These data led us to hypothesize that viral mRNAs are resistant to the nsp1-induced endonucleolytic RNA cleavage. To test this hypothesis, poly(A) containing intracellular RNAs were purified from SCoV-infected cells and incubated with GST, nsp1, or nsp1-mt in RRL. The RNAs were then extracted and subjected to primer extension analysis by using a 5′-end labeled primer that binds to a region ∼120 nt from the 5′-end of SCoV mRNA 9, the smallest and most abundant viral mRNA encoding the N protein. Due to the 3′ co-terminal nested structure of coronavirus mRNAs, it was predicted that this primer should bind to all 9 different SCoV mRNAs and generate primer extension products from all viral mRNAs. The expected sizes of the full-length primer extension products of viral mRNA 1 to mRNA 8 would exceed 300 nt, and our primer extension conditions were not suitable for precisely detecting potential nsp1-induced endonucleolytic RNA cleavage sites in these viral mRNAs. Hence, we examined whether nsp1 induced endonucleolytic RNA cleavage in mRNA 9, for which the expected length of full-length cDNA product was ∼120 nt. Remarkably, we did not detect primer extension premature termination signals that were specific for the sample incubated with nsp1 (Figure 9A). Furthermore, the amount of the full-length cDNA product of mRNA 9 was similar among these three samples. These data demonstrated that SCoV mRNA 9 was not susceptible to nsp1-mediated endonucleolytic RNA cleavage. To exclude an unlikely possibility that nsp1 exerts modification at the 5′-region of SCoV mRNAs in infected cells and cannot further modify viral mRNAs in RRL, we repeated the experiments by using mRNAs from cells infected with SCoV-mt. SCoV-mt encodes nsp1-mt [23] that does not induce modification of non-viral mRNAs [22], [23], and thus host and viral mRNAs in SCoV-mt-infected cells should not undergo the nsp1-induced endonucleolytic RNA cleavage. If SCoV mRNAs do not undergo the nsp1-induced endonucleolytic RNA cleavage in infected cells, then SCoV mRNA 9 and SCoV-mt mRNA 9 should have the same RNA sequence and structure. In the absence of nsp1, the size of the full-length primer extension product of SCoV-mt mRNA 9 and that of SCoV mRNA 9 was the same (Figure 9B). Similar to the results observed when SCoV mRNA 9 was unaffected in the presence of nsp1, incubation of SCoV-mt mRNA 9 with nsp1 did not generate premature primer extension termination signals. These data excluded the possibility that nsp1 induces endonucleolytic RNA cleavage near the 5′-region of SCoV mRNAs in infected cells and further support the conclusion that SCoV mRNA 9 is resistant to nsp1-mediated endonucleolytic RNA cleavage. We also performed primer extension analysis of mRNA 3 from SCoV-infected cells by using a primer that binds to a region about 150 nt from the 5′-end of mRNA 3 (Figure 9C). The nsp1-treated sample showed neither a reduced amount of the full-length cDNA product nor specific premature primer extension termination products, demonstrating that nsp1 did not induce endonucleolytic RNA cleavage in SCoV mRNA 3.

**Figure 10:**
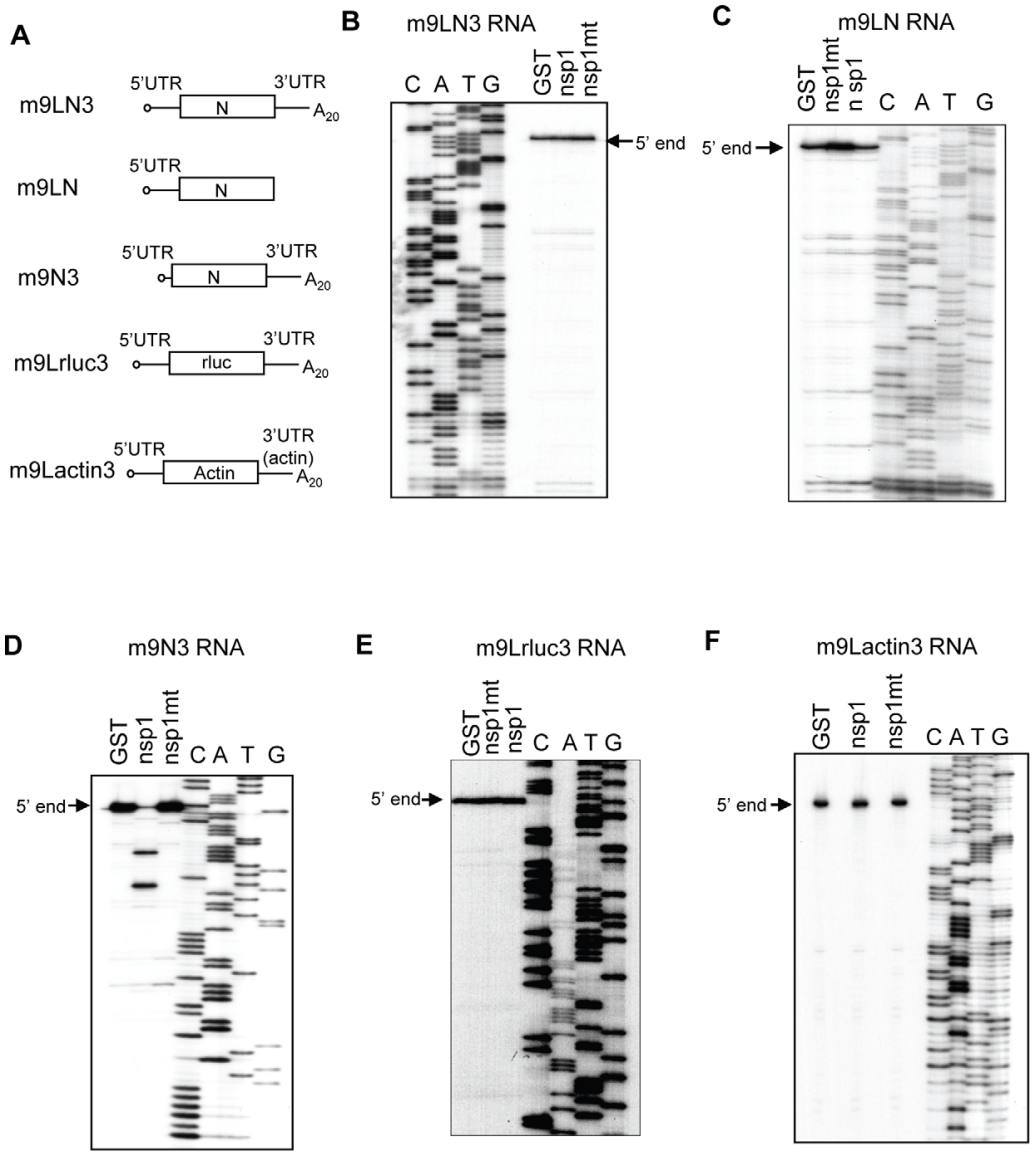
Susceptibilities of SCoV-like mRNAs to nsp1-induced RNA modification. (A) Schematic diagram showing the structures of SCoV mRNA 9-like m9LN3, m9LN, m9N3 and m9Lrluc3 RNAs. N, N ORF; rluc, rluc gene. (B, C) *In vitro*-synthesized m9LN3 RNA and m9LN RNA were independently incubated with GST, nsp1 or nsp1-mt in RRL. RNAs were extracted and subjected to primer extension analysis by using the same primer described in Figure 9A. 5′-end, full-length primer extension product. (D-F) The experiment was carried out using the same methods as in C, except that the following combinations of RNA and primer were used: (D) m9N3 RNA and a primer that binds ∼76 nt downstream of N gene translation initiation codon; (E) m9Lrluc3 RNA and a primer that binds ∼100 nt downstream of the rluc gene translation initiation codon; and (F) m9Lactin3 RNA template and a primer that binds ∼1 nt downstream of the β-actin gene translation initiation codon.

To identify the RNA element(s) in SCoV mRNAs that protects the viral mRNAs from the nsp1-induced endonucleolytic RNA cleavage, we prepared five different SCoV mRNA 9-like RNAs (Figure 10A). m9LN3 RNA has the following elements: a 5′-end cap structure, the authentic 5′ UTR of SCoV mRNA 9, carrying the common 72 nt-long leader sequence found in all SCoV mRNAs and an additional 8 nt sequence, the N gene ORF, the 3′ UTR, and a 20-nt long poly(A) sequence; naturally occurring SCoV mRNA 9 and m9LN3 are nearly identical in sequence, except that the coronavirus mRNAs have a longer 3′ poly(A) tail [53]. m9LN lacks the 3′ UTR and the 20 nt poly(A) sequence of m9LN3, while m9N3 lacks the 5′-end leader sequence of m9LN3. In m9Lrluc3, an rluc gene ORF is inserted in the place of the N gene ORF in m9LN3. In m9Lactin3, the 5′ UTR of actin mRNA is replaced with the 5′ UTR of SCoV mRNA 9; *in vitro*-synthesized actin mRNA served as a control. These RNAs were incubated with GST, nsp1 or nsp1-mt in RRL and subjected to primer extension analysis (Figures 10B-10F). Similar levels of the full-length primer extension products of expected sizes were detected in the m9LN3, m9LN, m9Lrluc3, and m9Lactin3 samples incubated with nsp1, GST or nsp1-mt (Figures 10B, 10C, 10E and 10F). We also did not detect any premature primer extension products in the nsp1-incubated samples. In contrast, incubation of m9N3 RNA with nsp1 resulted in the generation of two major premature primer extension products and a reduction in the levels of the full-length primer extension product (Figure 10D). As expected, nsp1 induced the endonucleolytic RNA cleavage in actin mRNA (Figure S3). These data showed that the presence of the leader sequence protected the viral mRNAs from the nsp1-induced endonucleolytic RNA cleavage.

**Figure 11:**
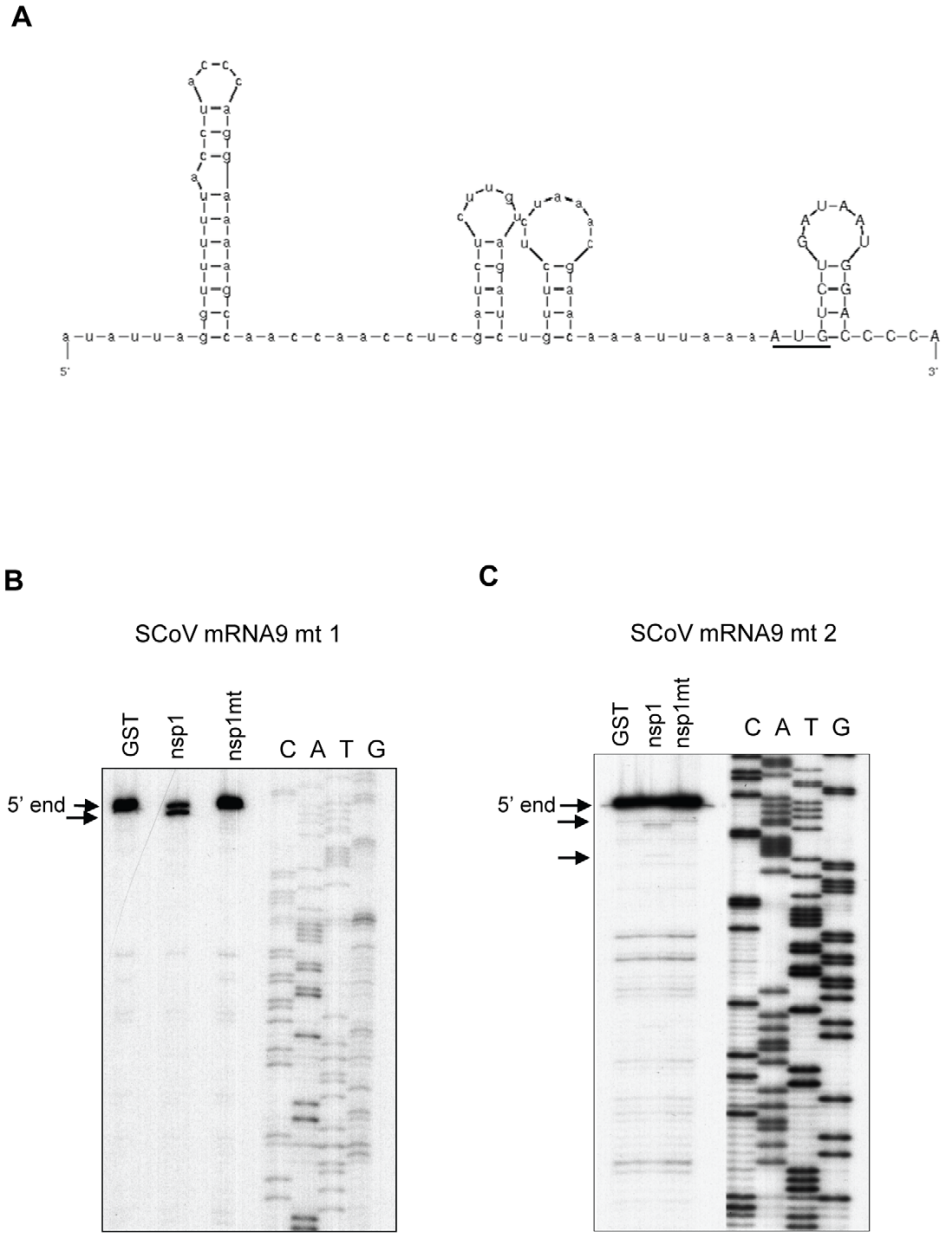
Susceptibilities of SCoV mRNA 9 mutants to nsp1-induced RNA modification. (A) Predicted secondary structure of the 5′ UTR of SCoV mRNA 9. Underlined AUG represents the translation initiation codon. (B, C) *In vitro*-synthesized SCoV mRNA 9 mt 1 and SCoV mRNA 9 mt 2 RNAs were independently incubated with GST, nsp1 or nsp1-mt in RRL. RNAs were extracted and subjected to primer extension analysis by using the same primer described in Figure 9A. 5′-end, full-length primer extension product. Arrows indicate premature primer extension products.

Next, we tested the importance of the 5′-terminal sequence of the SCoV leader in protecting viral mRNAs from the nsp1-induced endonucleolytic RNA cleavage. To this end, we generated two SCoV mRNA 9-derived mutants, SCoV mRNA 9 mt 1 and SCoV mRNA 9 mt 2. SCoV mRNA 9 mt 1 has an additional G at the 5′-end and SCoV mRNA 9 mt 2 carries a U to G mutation in the second nucleotide from the 5′-end. The 5′-end of SCoV mRNAs start with the sequence “m^7^GpppAUAU- - -”, while SCoV mRNA 9 mt 1 and SCoV mRNA 9 mt 2 start with “m^7^GpppGAUAU- - -” and “m^7^GpppAGAU- - -”, sequences, respectively (the additional G nucleotide and the mutated second nucleotide are underlined). A computer-assisted structural analysis showed that the 5′ UTR of SCoV mRNA 9 and the two mutants had the same RNA secondary structure (Figure 11A) and previous structural probing analysis confirmed the existence of three stem-loops within the leader sequence [54]. Nsp1 induced an endonucleolytic RNA cleavage in both mutants (Figures 11B and 11C), demonstrating the importance of an accurate and authentic 5′-terminal sequence of the SCoV leader in the protection of RNAs from the nsp1-induced endonucleolytic RNA cleavage.

To determine if nsp1 suppresses the translation of viral mRNAs, poly(A) containing RNAs were extracted from SCoV-infected or mock-infected cells and subjected to *in vitro* translation in RRL in the presence of nsp1, nsp1-mt, or GST. GLA mRNA served as a control. As shown in Figure S4A, the synthesis of the major viral protein, N, was substantially reduced in the presence of nsp1, but not GST or nsp1-mt, suggesting that nsp1 suppressed the translation of viral mRNAs in RRL. We could not examine the effect of nsp1 on translation of viral mRNAs in HeLa S10 extracts using a similar radiolabeling assay due to the relatively lower translational activity of HeLa S10 extracts compared to RRL (data not shown). Instead, we used a reporter assay to show that nsp1 suppressed the translation of the SCoV mRNA 9-like RNA, m9Lrluc3 RNA (Figure 10A), in the HeLa S10 extract (Figure S4B). These data showed that nsp1 did not induce endonucleolytic RNA cleavage in viral mRNAs but was able to suppress the translation of viral mRNAs *in vitro*.

## Discussion

The present study was aimed at identifying the nature of the nsp1-induced host mRNA modification, exploring the basis of the RNA modification site selection, and further understanding template-dependent, nsp1-induced RNA modification by examining several different RNA templates, including SCoV mRNAs and their derivatives.

### SCoV nsp1-mediated RNA modification

Nsp1 induced an endonucleolytic RNA cleavage within the IRES region of different dicistronic RNAs containing a picornavirus type I IRES or type II IRES [22] (Figure 2 and Figure S1). Incubation of cap-labeled ALA mRNA with nsp1 in RRL resulted in the generation of a cap-labeled RNA fragment 29 nt in length (Figure 7), demonstrating that nsp1 induced an endonucleolytic RNA cleavage in the ALA mRNA. Thus, the nature of the nsp1-mediated RNA modification is endonucleolytic RNA cleavage. This conclusion leads to the question as to the identity of the enzyme responsible for the endonucleolytic RNA cleavage. Based on the data that SCoV nsp1 has no similarities in its primary amino acid sequence or protein structure with any known host proteins, including RNases [55], and that binding of nsp1 to 40S ribosomal subunits is required for the nsp1-induced endonucleolytic RNA cleavage [22], we hypothesize that nsp1 is not an RNase, but uses a host RNase to induce endonucleolytic cleavage of template mRNAs that interact with 40S ribosomes. Several host endonucleases are involved in the mRNA surveillance pathways to detect stalls in translation and are known to exert their function in association with stalled ribosomes. The host cell RNase, SMG6, has been shown to play a central role in nonsense-mediated mRNA decay by inducing an endonucleolytic RNA cleavage in ribosome-associated host mRNAs containing a pre-mature termination codon [56]. The no-go mRNA decay pathway detects stalled ribosomes on mRNAs during translation elongation, and the yeast dom34/Hbs1 complex is involved in the endonucleolytic cleavage of mRNAs near the stalled site [57], [58]. The archaeal homolog of the yeast Dom34, Pelota, also exhibits an endonuclease activity [59]. The exosome is involved in the decay of nonstop mRNAs that lack a stop codon [60], and one of the subunits of the core eukaryotic exosome has endonuclease activity [61]-[63]. It is conceivable that nsp1, in association with ribosomes, uses one of the host endonucleases involved in mRNA surveillance pathways to induce RNA cleavage.

We identified the nsp1-induced RNA cleavage sites of several mRNAs. For Ren-EMCV-FF and Ren-PV-FF, RNA cleavage mainly occurred near the 3′-end of the IRES elements, where the 40S ribosome is recruited (Figures 2 and 3). Many nsp1-induced cleavage sites in capped mRNAs were mapped within 30 nt of the 5′ UTR (Figures 4, 5 and 6), and the ribosome footprint on an mRNA is about 28 nt in length [64], [65]. Thus the cleavage sites of template mRNAs were either in or proximal to the initial ribosome binding sites. From these data, we speculate that nsp1-induced RNA cleavage occurs as soon as a complex of nsp1, 40S ribosome, and translation initiation factors that bind to 40S ribosomes, e.g., eIF1, eIF1A, ternary complex, and eIF3 (we refer to this complex as the nsp1-40S complex) loads onto mRNA templates.

Gel electrophoresis of ^32^P cap-labeled ALA mRNA that had been incubated with nsp1 showed a discrete ∼29 nt ^32^P cap-labeled RNA fragment (Figure 7). Similarly, primer extension analysis of ALA mRNA that was incubated with nsp1 also showed a major premature termination site that corresponded to nucleotide 29 (site 3) (Figure 4B). Hence, two different experimental approaches convincingly demonstrated that the 29 nt-long RNA fragment was generated by nsp1-induced endonucleolytic RNA cleavage. Furthermore, the results of experiments using primer extension analysis of ALA mRNA (Figure 4) and gel electrophoresis analysis of cap-labeled ALA mRNA that underwent nsp1-induced RNA cleavage (Figure 7) led us to speculate that upon loading of the nsp1-40S complex onto ALA mRNA, an initial endonucleolytic RNA cleavage occurred at site 3 in ALA mRNA and the ALA mRNA transcripts that had undergone the initial RNA cleavage at site 3 were subjected to a second cleavage at site 4. RNA secondary structure modeling placed these sites in the vicinity of a stem-loop structure within the 5′ UTR (Figure 4D), which may imply that an RNase carrying out the initial RNA cleavage at site 3 could easily access site 4 and perform an additional RNA cleavage. However, the possibility of a host exonuclease further processing the endonucleolytically cleaved ALA mRNA at site 3 resulting in the generation of the primer extension termination site 4 cannot be excluded. Interestingly, the nsp1-induced RNA cleavage occurred between various dinucleotide sequences (Figures 3, 4, 5, 6 and 8), implying that the enzyme exerting endonucleolytic RNA cleavage exhibited little nucleotide preference.

### Template-dependent RNA cleavage of non-SCoV RNA induced by nsp1

Our previous and present studies showed that the nsp1-induced endonucleolytic RNA cleavage is template dependent. Nsp1 induced RNA cleavage within the 5′ UTR of capped mRNAs and within the picornavirus type I and type II IRES elements, while IRESes of HCV, CSFV, and CrPV were resistant to nsp1-induced RNA cleavage. There are differences in the requirement for translation initiation factors and mechanism of translation initiation among capped cellular mRNAs and mRNAs harboring different IRES elements. For cap-dependent translation initiation of host mRNAs, the 43S pre-initiation complex loads onto the 5′-region of mRNA through its interaction with the eIF4F, which binds to the 5′-end of mRNA and is formed by cap-binding eIF4E, eIF4A and eIF4G [30], [31]. Translation mediated by picornavirus type I and type II IRESes is independent of the cap-binding eIF4E, and it has been suggested that IRES-bound eIF4G and eIF4A recruit the 43S ribosome complex to viral mRNAs [37]. In contrast, HCV IRES-mediated translation starts by direct loading of the 40S ribosome onto the HCV IRES [30], [31] independent of any translation initiation factors. This is followed by the joining of eIF3 and the ternary complex to the HCV IRES-40S ribosome complex; it is believed that CSFV IRES-mediated translation initiation uses a similar mechanism [30], [31]. Only the 40S and 60S ribosomes, but not any translation initiation factors, are needed for translation initiation for the CrPV IRES [30].

Due to differences in the mechanism of translation initiation between capped cellular mRNAs and mRNAs with IRESes, several models are conceivable to explain the template-dependent nature of the nsp1-induced endonucleolytic RNA cleavage. One model proposes that eIF4A and eIF4G are required for nsp1-induced RNA cleavage. Namely, eIF4A and/or eIF4G when associated with mRNA templates may bind nsp1, which then induces activation of a putative RNase to carry out the RNA cleavage, and hence the absence of complex formation of HCV, CSFV or CrPV IRESes with eIF4A and eIF4G prevents the nsp1-induced RNA cleavage. Another model postulates that the nature of the complex between nsp1-40S ribosome and the IRESes of HCV, CSFV and CrPV and the resulting interface between the RNA and the 40S ribosome is such that it prevents the putative RNase from accessing and cleaving these IRESes. The third model proposes that binding of nsp1 to the 40S ribosome may induce a substantial conformational change in 40S ribosomes such that HCV, CSFV or CrPV IRESes may not be able to interact with the 40S-nsp1 complex. Thus, these latter mRNAs would not be in close proximity to the RNase activity induced by the formation of the nsp1-40S ribosome complex.

### Resistance of SCoV mRNAs from nsp1-induced RNA cleavage

The data that SCoV mRNA 3 and mRNA 9 were not susceptible to nsp1-mediated endonucleolytic RNA cleavage in RRL, along with the report that nsp1 did not suppress viral mRNA accumulation in infected cells [23], strongly suggest that SCoV mRNAs are resistant to nsp1-induced RNA cleavage in infected cells. *In vitro*-synthesized SCoV-like mRNAs containing the SCoV leader sequence were resistant to nsp1-induced RNA cleavage, while SCoV-like mRNA lacking the leader sequence was susceptible to the nsp1-induced RNA cleavage (Figures 9 and 10). Furthermore, the N gene ORF was dispensable for the resistance of virus-like mRNAs to nsp1-induced RNA cleavage (Figure 10), ruling out the possibility that N protein synthesized from SCoV mRNA 9 in RRL might have acted in *trans* to prevent the RNA cleavage. Thus, it was the leader sequence that protected viral and virus-like mRNAs from the nsp1-induced RNA cleavage.

Several mechanisms are conceivable as to how SCoV mRNAs escaped from the nsp1-induced endonucleolytic RNA cleavage. One is that the nsp1-40S complex loads onto SCoV mRNAs in such a way that the putative RNase that carries out the RNA cleavage cannot interact with SCoV mRNAs. Because nsp1 induced an RNA cleavage at the very 5′-end of SCoV mRNA 9 mt 1, carrying an extra G residue at the 5′-end of the transcripts, and SCoV mRNA 9 mt 2, carrying a U to G substitution at the second nucleotide position from the 5′-end, (Figure 11), the 5′-terminal region of the SCoV leader sequence may play a role in preventing the putative RNase from associating with the viral mRNAs. Alternatively, the binding of nsp1 to 40S ribosomes may induce structural alterations of the 40S ribosome such that interaction between nsp1-40S complex and SCoV mRNAs does not occur, and hence the putative RNase that carries out the nsp1-induced endonucleolytic RNA cleavage cannot gain access to the SCoV mRNAs. Like typical host mRNAs, SCoV mRNAs have a 5′ cap structure [6], [7], [52] and a 3′-end poly(A) tract. Also, preventing the interaction between eIF4E and eIF4G that inhibits cap-dependent translation also blocked coronavirus replication [66]; therefore, the SCoV mRNAs probably undergo cap-dependent translation. Accordingly, it is also possible that SCoV mRNAs and IRESes of HCV, CSFV or CrPV use different strategies to escape from the nsp1-induced RNA cleavage. For example, the leader sequence of SCoV mRNAs could recruit a host protein(s) to protect the viral mRNAs from RNA cleavage. Although SCoV mRNAs are resistant to nsp1-induced endonucleolytic RNA cleavage, translation of SCoV mRNAs and a reporter mRNA carrying the 5′ and 3′ UTRs of SCoV mRNA was suppressed in the presence of nsp1 (Figure S4). These data were not surprising, because nsp1 inactivates the translation functions of 40S ribosomes [22]. Although the stoichiometry between nsp1 and 40S ribosomes in SCoV-infected cells is unknown, the abundance of 40S ribosomal subunits most likely exceeds that of nsp1 in infected cells. SCoV mRNAs may be dissociated easily from the nsp1-40S complex or may not interact at all with the nsp1-40S complex and efficiently use the nsp1-free 40S ribosomes for their translation. In addition, there may be a mechanism whereby nsp1-40S ribosomes are excluded from sub-cellular locations where efficient viral translation takes place in infected cells to reduce the possibility that viral mRNAs interact with the nsp1-40S ribosome complex.

## Materials and Methods

### Preparation of poly A+ mRNAs from SCoV-infected cells and rabbit reticulocyte lysate

Vero E6 cells were infected with the Urbani strain SCoV or SCoV-mt [23] at a multiplicity of infection of 1. At 15 h post-infection, intracellular RNAs were extracted by using TRIzol reagent (Invitrogen). Total RNA was obtained from nuclease-untreated rabbit reticulocyte lysates (Promega) by proteinase K digestion, phenol/chloroform extraction and ethanol precipitation. In both samples, poly A+ mRNAs were further prepared by using Oligotex poly A^+^ RNA purification Kit (Qiagen).

### Plasmid construction

Replacing the rluc gene of pRL-SV40 (Promega) with the PCR product containing a T7 promoter sequence, 5′ UTR of human β-actin mRNA, rluc gene and a 50-nt-long poly(A) tail resulted in pALA-SV40 encoding ALA mRNA. A similar method was used to generate pGLA-SV40 encoding GLA mRNA, except that the 5′ UTR of human β-actin mRNA in pALA-SV40 was replaced by the 5′ UTR of rabbit β-globin mRNA. By using pGLA-SV40 as a template, we employed a QuikChange site-directed Mutagenesis Kit (Stratagene) to generate plasmids encoding GLA mRNA mutants. A reporter plasmid, pRL-HL, carrying in the following order: a CMV promoter, T7 promoter, rluc ORF, HCV IRES and fluc ORF, was described previously [22], [67]. The plasmids pRL-TMEV-FL, pRL-PV IRES-FL, pRL-CVB3 IRES-FL, pRL-HRV2 IRES-FL and pRL-CSFV IRES-FL were constructed by replacing the HCV IRES region of plasmid pRL-HL with that of following viruses and the nucleotide sequences of the viral genome from which they were derived: the DA strain of TMEV IRES, nt 395-1068; type 1 poliovirus IRES, nt 108-745; coxsackievirus B3 IRES, 100-741; human rhinovirus 2 IRES, nt 101-610; and classical swine fever virus IRES, nt 1-441. The RT-PCR product encoding rabbit β-globin mRNA was cloned into cloning vector pSMART (Lucigen), yielding pSG. The RT-PCR product of full-length SCoV mRNA 9 was cloned into pcDNA3.1 myc/His (Invitrogen), generating pcDm9LN3. The PCR product carrying a T7 class II Φ2.5 promoter, human β-actin mRNA (amplified from HEK 293 cells) and a 20-nt-long poly(A) tail was cloned into the vector pSMART, yielding pSA. The plasmid, pSm9Lactin3, encoding human β-actin mRNA carrying the 5′ UTR of SCoV mRNA 9, was generated by replacing the 5′ UTR of human β-actin mRNA in pSA with the 5′ UTR of SCoV mRNA 9. Sequence analyses of the plasmids confirmed the presence of the expected sequence.

### *In vitro* RNA transcription

Transcription of dicistronic RNA transcripts was described previously [22], [67]. Capped and polyadenylated RNA transcripts were synthesized from linearized plasmids *in vitro* by using the mMESSAGE mMACHINE T7 kit or T7 Ultra kit (Applied Biosystems) according to the manufacturer′s protocol. Synthesis of SCoV mRNA 9 was performed as previously described [68], [69]. The PCR product of full-length SCoV mRNA 9 was generated by using pcDm9LN3 as a template and primers T7 phi2.5-5′SARS, 5′*-*CGGAGTAATACGACTCACTATTATATTAGGTTTTTACCTACCC-OH, and SARS 3′ UTR primer, 5′-TTTTTTTTTTTTTTTTTTTTGTCATTCTCCTAAGAAG-OH; to ensure that the 5′-proximal sequence of the *in vitro* synthesized RNA was identical to that of authentic SCoV mRNA 9, the T7 class II Φ2.5 promoter (underlined sequence) was used to initiate transcription by ATP, instead of GTP [70]. To serve as a template for the synthesis of m9N3 RNA, SCoV mRNA 9 mt 1 RNA, and SCoV mRNA 9 mt 2 RNA, a PCR product was generated using the following forward primers with the SARS 3′ UTR primer, respectively: 5′-AATTAATACGACTCACTATAG AACAAATTAAAATGTCTGATA ATGG-OH (T7 promoter sequence underlined); 5′-AATTAATACGACTCACTATAG ATATTAGGTTTTTACCTACC-OH (T7 promoter sequence underlined); and 5′-CGGGATCCGAG TAATACGACTCACTATT AGATTAGGTTTTTACCTACCC-OH (the T7 class II Φ2.5 promoter sequence underlined). The amplicon, purified on an agarose gel, served as a template for the synthesis of uncapped SCoV mRNA 9 using the MEGAscript T7 kit (Applied Biosystems). The *in vitro* synthesized SCoV mRNA 9 was purified with the RNeasy mini kit (Qiagen), capped with vaccinia virus capping enzyme using the ScriptCap m^7^G Capping System (Epicentre Biotechnology) and further purified with the RNeasy mini kit (Qiagen). Similar procedures were used for the generation of m9LN and m9LrLuc3 RNAs, except that the PCR products, as shown in Figure 10, serve as templates for RNA synthesis, and a reverse primer that binds to the 3′-end of N gene was used to generate the PCR for transcription of m9LN RNA.

### *In vitro* translation

*In vitro* translation was performed using the Retic Lysate IVT kit (Applied Biosystems). RRL was initially incubated with 1 µg GST protein, nsp1 protein or nsp1-mt protein purified from *E. coli* at 4^o^C for 10 min [22]. Then, 0.25 µg mRNA and an amino acid mixture (Promega, to a final concentration of 1 mM) were added; the molar ratio of nsp1 to mRNA was approximately 200∶1. Samples were incubated at 30°C for 10 min. In some experiments, HeLa S10 extract was added to RRL at a final concentration of 20% [71], as indicated, and translation was performed at 30°C for 10 min. After incubation, the samples were incubated with proteinase K, and the extracted RNAs were subjected to Northern blot or primer extension analyses. In some experiments, luc assays were performed by adding 5 µl translation product in 100 µl Renilla Luciferase Lysis Buffer (Promega). Luminescence was measured by using a Renilla luciferase assay system (Promega).

### Primer extension analysis

After incubation of RNA samples with the 5′-end ^32^P labeled primers (40,000 c.p.m), primer extension was performed by using the Primer Extension System (Promega) according to the manufacturer's protocol. The RNAs, primer sequence, and primer-binding site and conditions are as follows: GLA and ALA mRNAs, 5′-TTTTTCTGAATCATAATAATTAA-3′, ∼100 nt downstream of rluc translation initiation codon, incubation at 42°C for 1 h and subsequent incubation at room temperature for 10 min; Ren-EMCV-FF, 5′-AGCAATTGTTCCAGGAACCAGGG, ∼100 nt downstream of fluc translation initiation codon, incubation at 55°C for 1 h and subsequent incubation at room temperature for 10 min; Ren-PV-FF, 5′-GGGCCTTTCTTTATGTTTTTGGCG, ∼6 nt downstream of AUG translation initiation codon of fluc gene, incubation at 55°C for 1 h and subsequent incubation at room temperature for 10 min; SCoV mRNA 9, m9LN3, and m9LN, 5′-GGGTCCACCAAATGTAATGC-3′, ∼44 nt downstream of N gene translation initiation codon, incubation at 50°C for 1 h and subsequent incubation at room temperature for 10 min; SCoV mRNA 3, 5′-TGTAGCATGAACAGTACTTGC-3′, ∼75 nt downstream of 3a gene translation initiation codon, incubation at 50°C for 1 h and subsequent incubation at room temperature for 10 min; m9LrLuc3, 5′- TTTTTCTGAATCATAATAATTAA-3′, ∼100 nt downstream of rluc translation initiation codon, incubation at 42°C for 1 h and subsequent incubation at room temperature for 10 min; and m9N3, 5′-GTCCTCCATTCTGGTTATTGTC-3′, ∼76 nt downstream of N gene translation initiation codon, incubation at 50°C for 1 h and subsequent incubation at room temperature for 10 min; m9Lactin3 and actin, 5′- AGCGCGGCGATATCATCATC-3′, ∼1 nt downstream of the AUG translation initiation codon of β-actin gene, incubation at 55°C for 1 h and subsequent incubation at room temperature for 10 min. AMV reverse transcriptase was used for primer extension. After ethanol precipitation, the primer extension products were resolved in 8% polyacrylamide/7M Urea DNA sequencing gels.

### Analysis of the nsp1-induced cleavage product of cap-labeled ALA mRNA and preparation of RNA size markers

Uncapped and polyadenylated ALA mRNA was prepared by using the MEGAscript *in vitro* transcription kit (Applied Biosystems). Cap labeling was performed by incubation of 30 µg of uncapped RNA with vaccinia virus capping enzyme (ScriptCap m^7^G capping system) in the presence of α-^32^P GTP (3,000 Ci/mmole, MP) at 37°C for 1 h. Cap-radiolabeled ALA mRNA was purified with the RNeasy mini kit and 0.75 µg RNA (approximately 100,000 cpm/µg) was used for *in vitro* translation in RRL. Then the sample was incubated with proteinase K, and RNA was extracted by phenol/chloroform.

The RNA size marker (ranging from 10 nt to 150 nt) was prepared with the Decade Marker system (Applied Biosystems) according to the manufacturer's instruction with modifications. Full-length Decade Marker RNA (0.5 µg) of 150 nt in length was cap labeled with vaccinia virus capping enzyme in the presence of α-^32^P GTP (3,000 Ci/mmole, MP). After ethanol precipitation, the cap-labeled marker RNA was dissolved in a solution containing 8 µl H_2_O, 1 µl 10X kinase buffer (Decade Marker system, Applied Biosystems) and 1 µl 10X cleavage reagent (Decade Marker system, Applied Biosystems). The full-length RNA marker was cleaved into an RNA ladder by incubation at room temperature for 5 min. The produced RNA ladder contained a set of cap-labeled RNA molecular weight markers of 150, 100, 90, 80, 70, 60, 50, 40, 30, 20 and 10 nt in length. An equal volume of 2x proteinase K digestion buffer was added to the prepared Decade Marker RNA ladder to adjust the salt concentration to a level similar to that of RNA samples extracted from RRL. The RNA samples were analyzed on 10% polyacrylamide/7M Urea DNA sequencing gels.

## Supporting Information

Figure S1 **Susceptibilities of Ren-CVB-FF and Ren-HRV2-FF to nsp1-mediated RNA cleavage.** (A) Schematic diagram of the structures of full- length dicistronic RNA transcripts (Full length), RNA 1 containing the 5′ rluc gene and intercistronic IRES sequence and RNA 2 containing only the 5′ rluc gene. (B and C) Ren-CVB-FF (B) and Ren-HRV2-FF (C) were independently incubated with GST, nsp1, or nsp1-mt or without any protein (mock) in RRL+HeLa at 30°C for 10 min. RNA samples were extracted and analyzed by Northern blotting using an 5′ rluc probe (left panel) and 3′ fluc probe (right panel). Marker represents a mixture of *in vitro*-transcribed full length RNA transcripts, RNA 1 and RNA 2.
(TIF)

Figure S2 **Susceptibilities of discistonic RNA transcripts carrying different IRESes to nsp1-mediated RNA cleavage in cells.** Plasmids pCAGGS-CAT encoding the CAT gene, pCAGGS-nsp1 encoding nsp1 and pCAGGS-nsp1 mt encoding nsp1-mt were co-transfected with each of the CMV promoter-driven dicistronic reporter plasmids encoding the 5′ rluc gene, IRES and 3′ fluc gene in HEK293 cells. The IRESes included in the RNA expression plasmid were: TMEV IRES (A), poliovirus IRES (B), HCV IRES (C) or CSFV IRES (D). After 24 hr, total RNA was extracted from the cells by using Trizol reagent. Samples were treated with DNase I and RNAs were purified with RNeasy (Qiagen). The RNAs were subjected to Northern blot analysis using an rluc probe.
(TIF)

Figure S3 **Susceptibility of actin mRNA to nsp1-induced RNA modification.** *In vitro*-synthesized actin mRNA was incubated with GST, nsp1 or nsp1-mt in RRL. RNAs were extracted and subjected to primer extension analysis by using the same primer described in Figure 10F. 5′-end, full-length primer extension product. Arrows indicate premature primer extension products.
(TIF)

Figure S4 **Effect of nsp1 on translation of SCoV mRNA in RRL and SCoV-like mRNA encoding a reporter protein in HeLa S10 extract.** (A) Intracellular polyA+ RNAs obtained from SCoV-infected Vero E6 cells (SCoV-infected) were subjected to an *in vitro* translation reaction in the presence of ^35^S-methionine along with GST, nsp1 or nsp1-mt in RRL (left panel). As a control, intracellular poly A+ RNA from mock-infected Vero E6 cells was subjected to *in vitro* translation in the presence of GST (Mock). A similar *in vitro* translation assay was performed using *in vitro*-transcribed capped GLA mRNA (right panel). The radiolabeled proteins were analyzed by SDS-PAGE and autoradiography. SCoV major structural protein (N protein) and rluc protein are indicated by arrows. (B) *In vitro* synthesized m9Lrluc3, a SCoV mRNA 9-like reporter mRNA carrying an rluc gene in the place of N gene ORF (Figure 10), was subjected to an *in vitro* translation assay in HeLa S10 extract in the presence of GST (GST) or nsp1 proteins (nsp1). After 1.5 h incubation, the rluc activity was measured and represented as the average of three independent experiments (+/- SD).
(TIF)
