# Supplementary figures and images for: SARS Coronavirus nsp1 Protein Induces Template-Dependent Endonucleolytic Cleavage of mRNAs: Viral mRNAs Are Resistant to nsp1-Induced RNA Cleavage
Source: PLoS Pathog. 2011 Dec 8;7(12):e1002433. doi: 10.1371/journal.ppat.1002433 (PMC3234236; doi:10.1371/journal.ppat.1002433)

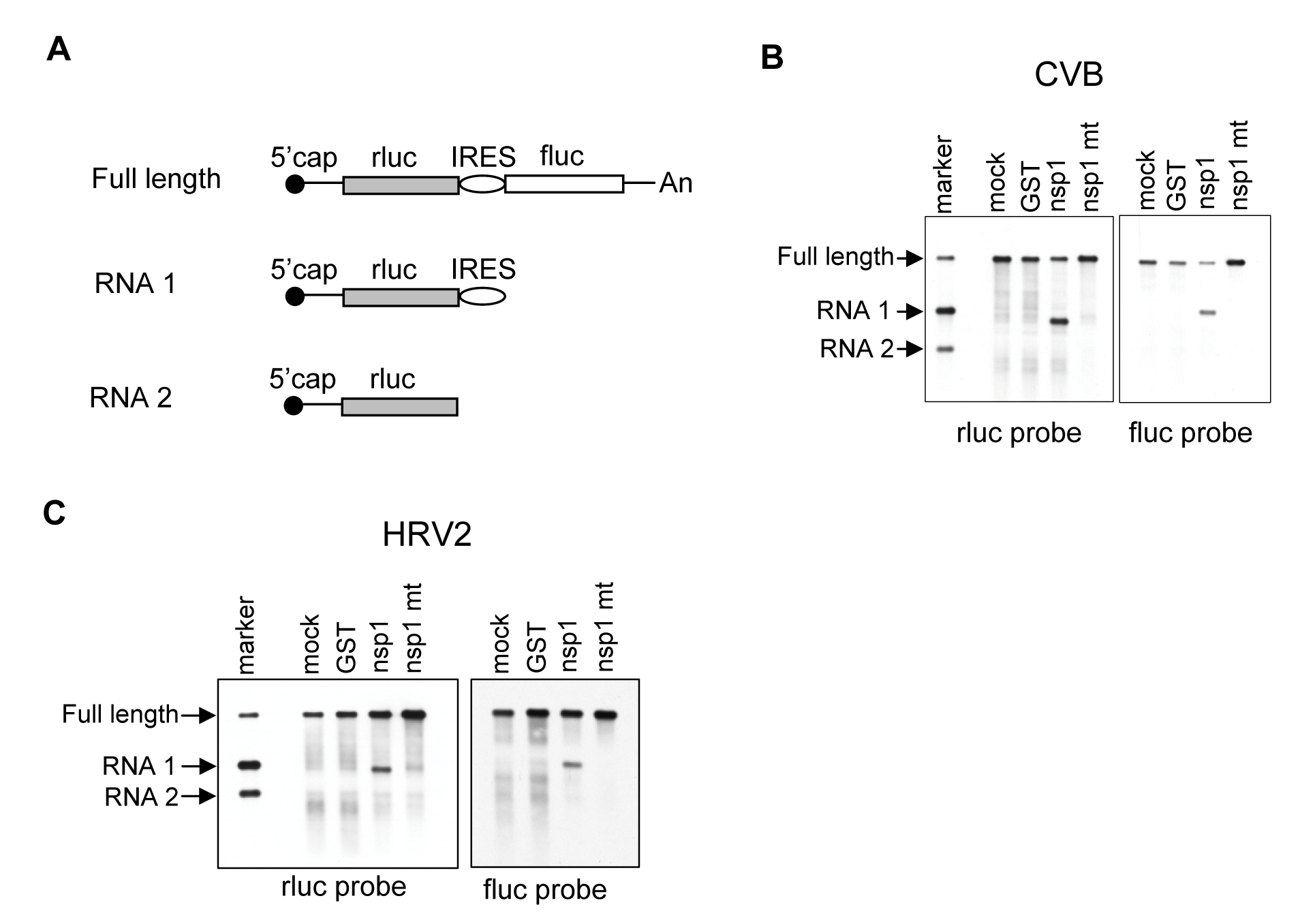

Supplement: Figure S1 — Susceptibilities of Ren-CVB-FF and Ren-HRV2-FF to nsp1-mediated RNA cleavage. (A) Schematic diagram of the structures of full- length dicistronic RNA transcripts (Full length), RNA 1 containing the 5′ rluc gene and intercistronic IRES sequence and RNA 2 containing only the 5′ rluc gene. (B and C) Ren-CVB-FF (B) and Ren-HRV2-FF (C) were independently incubated with GST, nsp1, or nsp1-mt or without any protein (mock) in RRL+HeLa at 30°C for 10 min. RNA samples were extracted and analyzed by Northern blotting using an 5′ rluc probe (left panel) and 3′ fluc probe (right panel). Marker represents a mixture of in vitro-transcribed full length RNA transcripts, RNA 1 and RNA 2. (TIF) [file ppat.1002433.s001.tif]

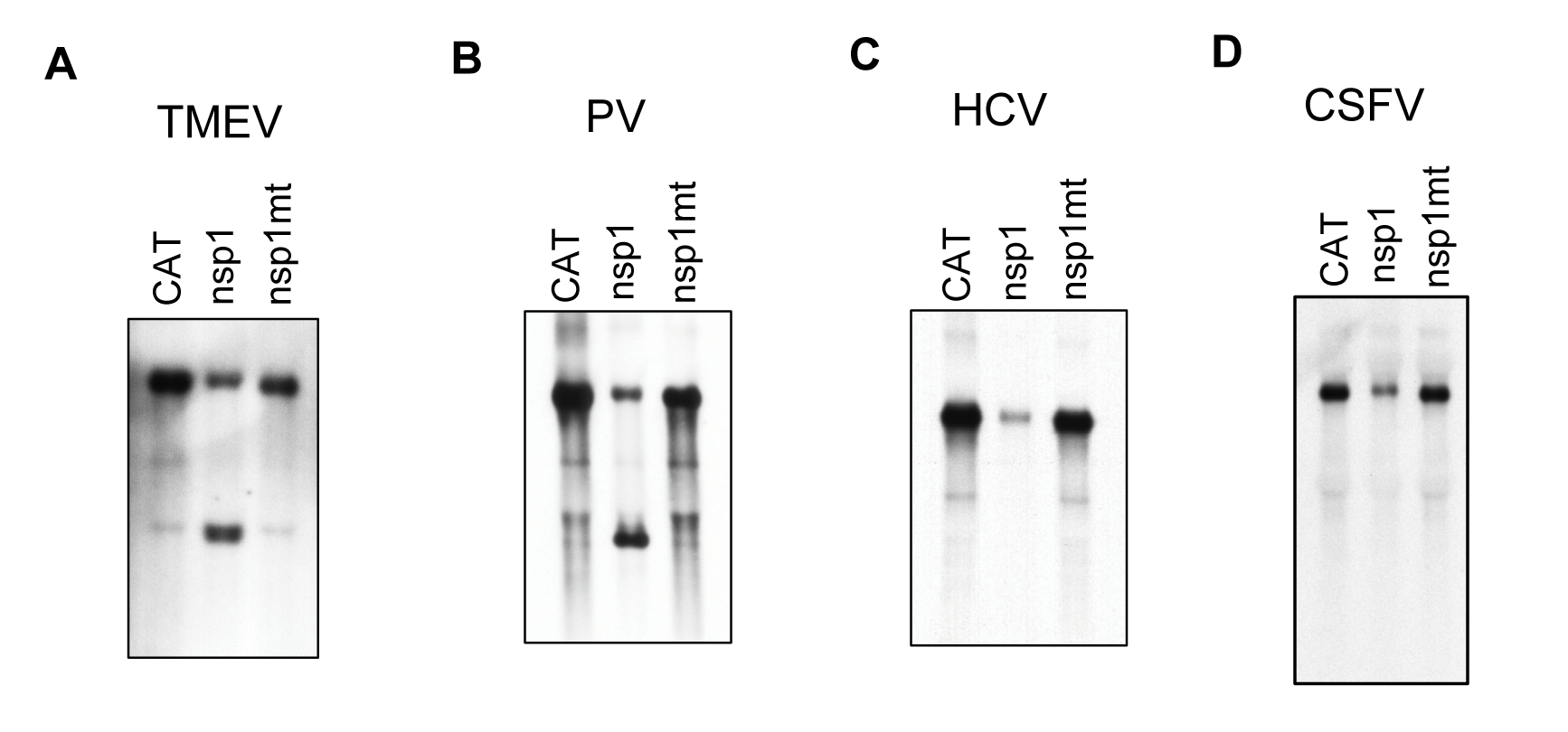

Supplement: Figure S2 — Susceptibilities of discistonic RNA transcripts carrying different IRESes to nsp1-mediated RNA cleavage in cells. Plasmids pCAGGS-CAT encoding the CAT gene, pCAGGS-nsp1 encoding nsp1 and pCAGGS-nsp1 mt encoding nsp1-mt were co-transfected with each of the CMV promoter-driven dicistronic reporter plasmids encoding the 5′ rluc gene, IRES and 3′ fluc gene in HEK293 cells. The IRESes included in the RNA expression plasmid were: TMEV IRES (A), poliovirus IRES (B), HCV IRES (C) or CSFV IRES (D). After 24 hr, total RNA was extracted from the cells by using Trizol reagent. Samples were treated with DNase I and RNAs were purified with RNeasy (Qiagen). The RNAs were subjected to Northern blot analysis using an rluc probe. (TIF) [file ppat.1002433.s002.tif]

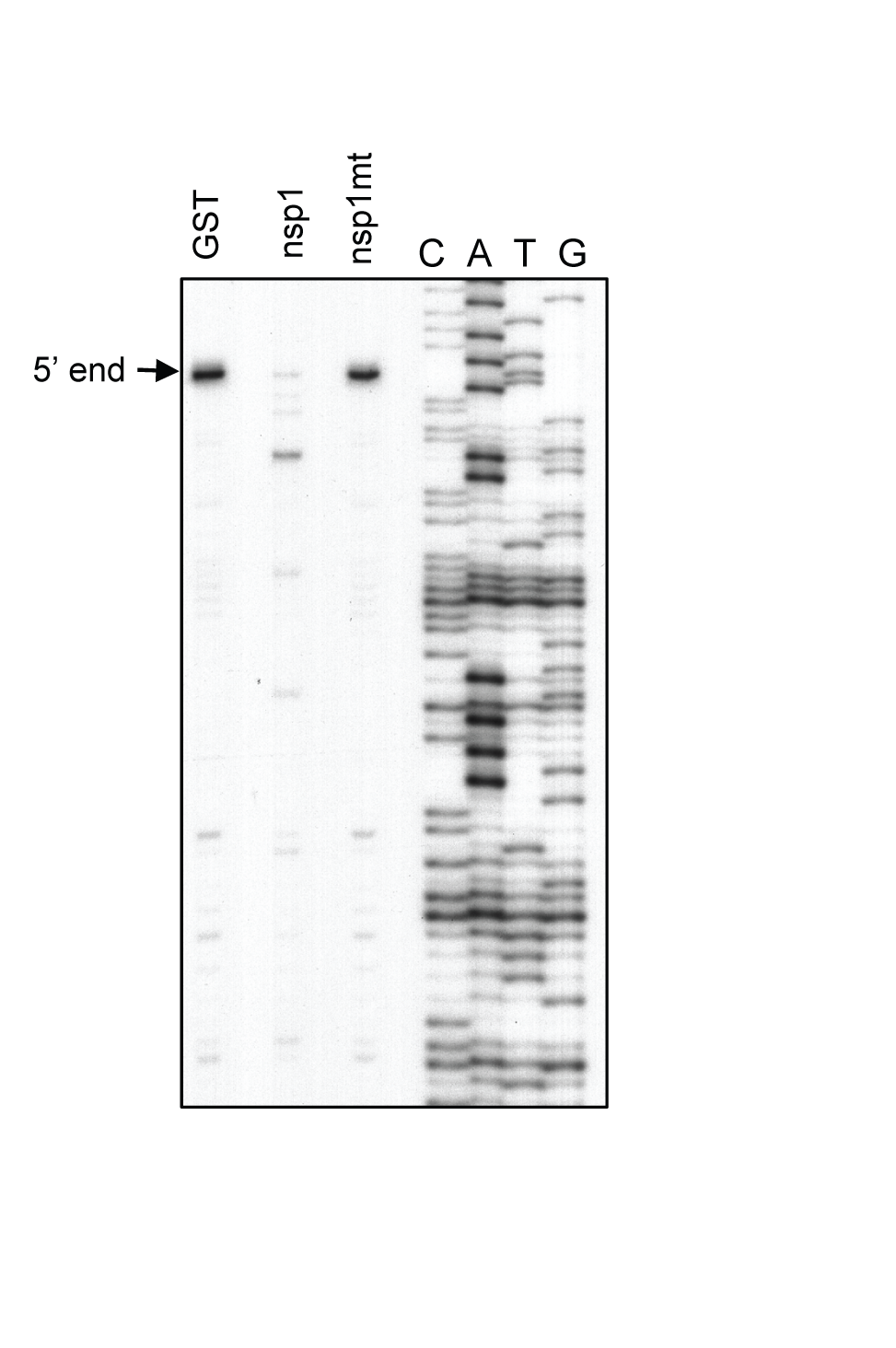

Supplement: Figure S3 — Susceptibility of actin mRNA to nsp1-induced RNA modification. In vitro-synthesized actin mRNA was incubated with GST, nsp1 or nsp1-mt in RRL. RNAs were extracted and subjected to primer extension analysis by using the same primer described in Figure 10F. 5′-end, full-length primer extension product. Arrows indicate premature primer extension products. (TIF) [file ppat.1002433.s003.tif]

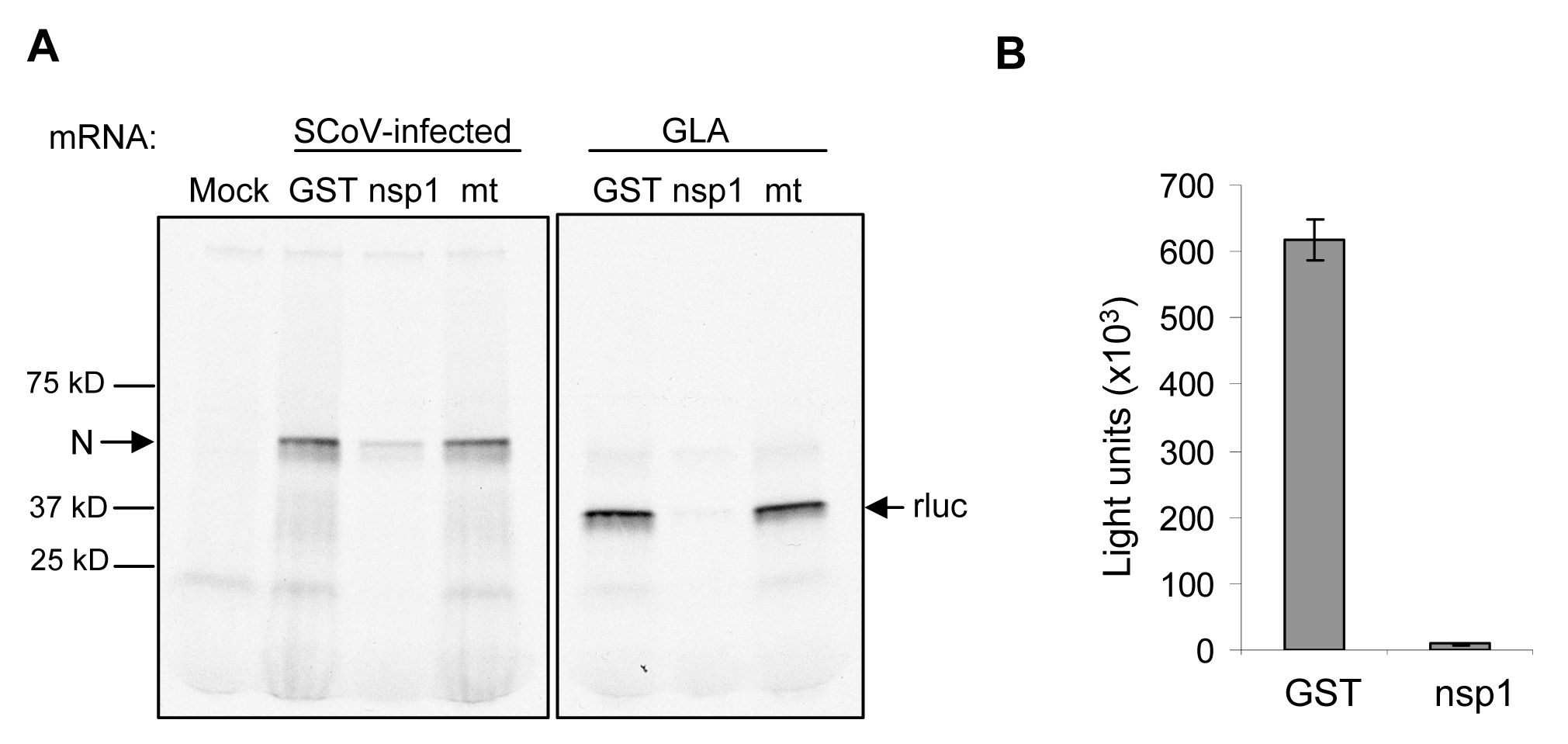

Supplement: Figure S4 — Effect of nsp1 on translation of SCoV mRNA in RRL and SCoV-like mRNA encoding a reporter protein in HeLa S10 extract. (A) Intracellular polyA+ RNAs obtained from SCoV-infected Vero E6 cells (SCoV-infected) were subjected to an in vitro translation reaction in the presence of 35S-methionine along with GST, nsp1 or nsp1-mt in RRL (left panel). As a control, intracellular poly A+ RNA from mock-infected Vero E6 cells was subjected to in vitro translation in the presence of GST (Mock). A similar in vitro translation assay was performed using in vitro-transcribed capped GLA mRNA (right panel). The radiolabeled proteins were analyzed by SDS-PAGE and autoradiography. SCoV major structural protein (N protein) and rluc protein are indicated by arrows. (B) In vitro synthesized m9Lrluc3, a SCoV mRNA 9-like reporter mRNA carrying an rluc gene in the place of N gene ORF (Figure 10), was subjected to an in vitro translation assay in HeLa S10 extract in the presence of GST (GST) or nsp1 proteins (nsp1). After 1.5 h incubation, the rluc activity was measured and represented as the average of three independent experiments (+/- SD). (TIF) [file ppat.1002433.s004.tif]
